# Identification of scaffold proteins for improved endogenous engineering of extracellular vesicles

**DOI:** 10.1038/s41467-023-40453-0

**Published:** 2023-08-07

**Authors:** Wenyi Zheng, Julia Rädler, Helena Sork, Zheyu Niu, Samantha Roudi, Jeremy P. Bost, André Görgens, Ying Zhao, Doste R. Mamand, Xiuming Liang, Oscar P. B. Wiklander, Taavi Lehto, Dhanu Gupta, Joel Z. Nordin, Samir EL Andaloussi

**Affiliations:** 1 Division of Biomolecular and Cellular Medicine, Department of Laboratory Medicine, Karolinska Institutet, Huddinge, Sweden; 2 Institute of Technology, University of Tartu, Tartu, Estonia; 3 Department of Hepatobiliary Surgery, Shandong Provincial Hospital Affiliated to Shandong First Medical University, Jinan, China; 4 Institute for Transfusion Medicine, University Hospital Essen, University of Duisburg-Essen, Essen, Germany; 5 Clinical Research Center, Karolinska University Hospital, Stockholm, Sweden

**Keywords:** Nanoparticles, Protein delivery, Therapeutics, Assay systems, Drug delivery

## Abstract

Extracellular vesicles (EVs) are gaining ground as next-generation drug delivery modalities. Genetic fusion of the protein of interest to a scaffold protein with high EV-sorting ability represents a robust cargo loading strategy. To address the paucity of such scaffold proteins, we leverage a simple and reliable assay that can distinguish intravesicular cargo proteins from surface- as well as non-vesicular proteins and compare the EV-sorting potential of 244 candidate proteins. We identify 24 proteins with conserved EV-sorting abilities across five types of producer cells. TSPAN2 and TSPAN3 emerge as lead candidates and outperform the well-studied CD63 scaffold. Importantly, these engineered EVs show promise as delivery vehicles in cell cultures and mice as demonstrated by efficient transfer of luminal cargo proteins as well as surface display of different functional entities. The discovery of these scaffolds provides a platform for EV-based engineering.

## Abstract (web summary)

Extracellular vesicles are naturally occurring nanoparticles that are gaining ground as delivery modalities for therapeutics. Here, the authors conducted a large-scale screening programme to identify potential scaffold proteins for cargo loading into extracellular vesicles.

## Introduction

Extracellular vesicles (EVs) are membrane-enclosed particles that are secreted by most types of cells^1–3^. By harboring diverse macromolecules, EVs are important mediators of intercellular communication owing to their intrinsic tropism and protection of their luminal contents from rapid degradation. In combination with favorable safety profiles, EVs have gained tremendous attention as a potential next-generation therapeutic modality for a wide range of diseases^4,5^.

Harnessing EVs for therapeutic applications relies primarily on their contents. In the simplest scenario, EVs are inherently packed with therapeutic molecules from their source cell^6–9^. For instance, mesenchymal stem cell-derived EVs have repeatedly been shown to reflect the regenerative and immunomodulatory properties of their parental cells^10^. EVs can also be deliberately loaded with molecules of interest through exogenous or endogenous means. Exogenous loading is performed on pre-isolated EVs using physical methods such as sonication, electroporation or chemical conjugation. This approach is largely restricted to small payloads including miRNAs and low molecular weight chemicals and is associated with technical challenges related to RNA precipitation and physical impairment or aggregation of EVs^11,12^. Larger payloads like proteins are often loaded endogenously during EV biogenesis in producing cells^13–15^. Typically, producer cells are genetically instructed to overexpress the protein of interest fused to an EV-sorting protein, thereby boosting endogenous sorting of the cargo protein. This strategy can direct molecules to the surface or the lumen of EVs^16^. In contrast to surface display, luminal loading prevents premature dissociation/degradation of the cargo and is therefore the approach of choice for molecules that are prone to degradation and operate in the cytosol or nucleus of recipient cells. We and others have demonstrated the applicability of endogenous loading by incorporating protein therapeutics like super-repressor IκB and receptor protein decoys into or onto EVs^17–22^. Additionally, this approach allows for indirect loading of RNA therapeutics by fusion of RNA-binding proteins to EV-sorting proteins^23–26^.

Although a versatile strategy, endogenous loading is essentially determined by the abundance of the sorting protein in an EV population. This refers not only to the levels it can reach per EV, but more importantly to its presence across different EV subpopulations. Generally, EV populations are heterogenous pools that to date the field struggles to characterize and physically separate into distinct subpopulations^27,28^, which complicates endogenous loading strategies. On the upside, 213 proteins were found to be conserved across EVs from 60 different cell types from the National Cancer Institute (NCI-60), therefore lending themselves as potential EV-sorting candidates^29^. However, until now only a few proteins have been well-characterized for loading proteins into EVs. Most of these are multi-pass transmembrane proteins belonging to the tetraspanin superfamily, such as CD9, CD63 and CD81^30^. In reflection of the heterogeneity of EVs, overexpression of CD63-GFP fusion protein resulted in 51% GFP-positive particles at most^31–33^. Efforts have been made to identify alternative EV-sorting candidates showing promise for PTGFRN and BASP1^32^ as well as TSPAN14^33^. These studies focused on low-throughput, GFP-centered quantification methods and included a maximum of 14 candidate proteins.

Here, we conduct a large-scale comparative study including 244 potential candidates to obtain a more comprehensive picture and in the hopes of identifying other EV-sorting proteins, Throughout the entire study, TSPAN2, TSPAN3 and CD63 consistently emerge as highly efficient EV-sorting proteins with robust luminal loading ability across different producer cell types. Furthermore, TSPAN2- and TSPAN3-engineered EVs show potential as delivery modalities not only ascertained by efficient uptake in vitro and in vivo but also owing to ample engineering possibilities (i.e., surface display and luminal cargo loading). Therefore, we believe that this discovery provides a steppingstone for endogenous engineering approaches to load cargo into or onto EVs, thereby enabling potential therapeutic applications.

## Results

### A simple assay for screening EV-sorting proteins

**Fig. 1:**
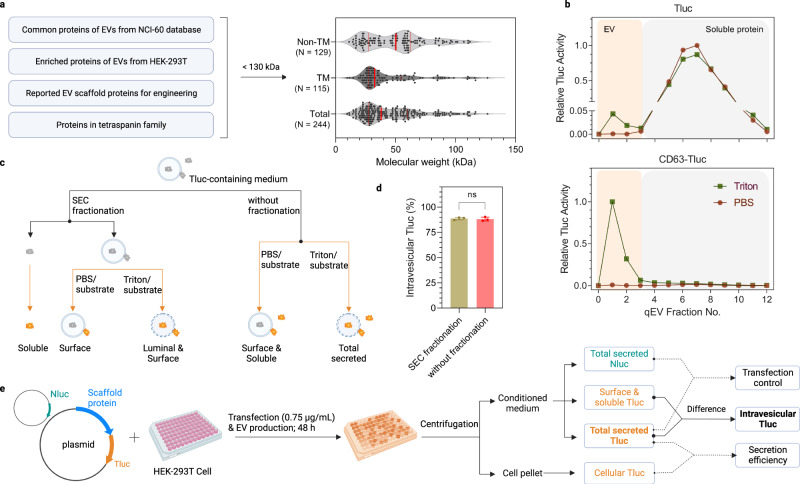
A bioluminescence screening protocol for quantification of luminal cargo proteins in EVs. **a** Selection criteria and overview of EV-sorting protein candidates. The red solid lines indicate the 25%, 50% and 75% percentile values. **b** SEC elution profiles of conditioned media from HEK-293T cells expressing Tluc or CD63-Tluc. Tluc activity in each fraction was quantified directly (group PBS) or after membrane lysis (group Triton) and normalized to the fraction with the highest signal. EVs and soluble proteins were recovered in fractions 0-3 and 4–12, respectively. **c** Scheme of differentiating Tluc forms in conditioned media. **d** Percentage of intravesicular Tluc for CD63-Tluc using fractionated and unfractionated media. Results are shown as the mean ± standard deviation of three biological replicates. Two-sided Student’s *t* test (*P* > 0.9999). ns: not significant. **e** Outline of the screening procedure and data analyses. HEK-293T cells were grown in 96-well microplates and co-transfected with Tluc fusion plasmid and Nluc plasmid. Cell cultures were centrifuged and Tluc activity was measured in the cell pellet and conditioned media. Nluc activity was only quantified in the conditioned media. **c**, **e** Created with BioRender.com. Source data are provided as a Source Data file. SEC size exclusion chromatography.

In search of efficient EV-sorting proteins, a list of candidates was compiled based on literature review and proteomics databases. Potential candidates were derived from either (1) proteins found to be enriched in EVs across the NCI-60 cells^29^, (2) proteins abundant in EVs produced by human embryonic kidney epithelial (HEK)-293T cells^34^, (3) reported EV-sorting proteins as references to previous studies^32,33^, and (4) all proteins in the tetraspanin superfamily. Proteins larger than 130 kDa were excluded to facilitate overexpression/engineering. A total of 244 candidates with a median size of 38 kDa were included, of which 129 were non- and 115 were transmembrane proteins (Fig. 1a; see the Data Source file for a complete list).

To assess the luminal loading ability of the candidates into EVs, we developed an assay based on the luciferase reporter ThermoLuc (Tluc; 60.5 kDa)^18^. In brief, Tluc was fused to the C termini of all candidates, bearing in mind that N termini are usually the site for signal peptides and post-translational modifications. The plasmids encoding the fusion proteins were transfected into HEK-293T cells. After 48 h, the conditioned media were collected and further processed prior to bioluminescence measurements. Initially, to evaluate the feasibility of this assay, the conditioned media of cells expressing Tluc alone and CD63-Tluc were analyzed. Both were fractionated with size exclusion chromatography (SEC) columns to separate vesicles from free proteins (Fig. 1b)^35^. The fractions were treated either with PBS to determine soluble/surface-associated Tluc or the detergent Triton X-100 to detect total secreted Tluc (Fig. 1c). Compared to Tluc alone, fusion with CD63 resulted in a prominent shift of Tluc towards the EV fraction (Fig. 1b). Notably, Tluc activity in the EV fractions was only detected upon membrane lysis, indicating that Tluc substrate is unable to cross the EV membrane and react with luminal luciferase. This implies that SEC fractionation is dispensable for quantifying luminal proteins. This is further supported by comparison of unfractionated and fractionated media of CD63-Tluc-expressing cells, which revealed no significant differences in the percentage of intravesicular Tluc (Fig. 1d). Taken together, these data show that this assay can be used in a high-throughput format to identify potential EV-loading scaffolds.

Moving forward, the principle for screening all 244 candidates was the same as above. For downstream analyses, the proteins were primarily evaluated on the absolute amount of intravesicular Tluc, derived from the difference in Tluc signal detected with and without membrane lysis, or the relative percentage thereof (Fig. 1e). Information on fusion protein expression was obtained by measuring Tluc in the EV-producing cells. Additionally, when specified, the data was normalized to a transfection control in the form of a plasmid encoding NanoLuc (Nluc) luciferase that was spiked into the transfection mixture to account for possible transfection variations.

### Screening identifies dozens of EV-sorting proteins

**Fig. 2:**
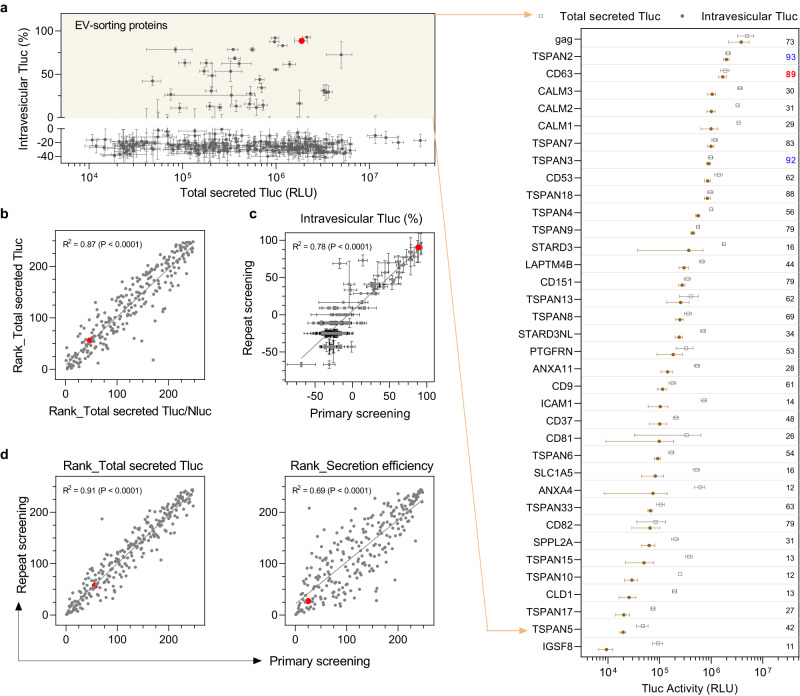
EV-sorting ability of candidate proteins. **a** Overview of all 244 candidates plotting total secreted Tluc against percentage of intravesicular Tluc. EV-sorting proteins were defined to have a percentage of intravesicular Tluc above zero (yellow area) and are shown in the grouped dot plot. The value refers to the percentage of intravesicular Tluc. Proteins are marked with gene names. **b** Correlation between the rank regarding total secreted Tluc and the rank regarding total secreted Tluc/Nluc ratio. **c** Correlation of the percentage of intravesicular Tluc obtained from the primary and repeat screening. **d** Correlation of the rank regarding secreted Tluc or secretion efficiency between the primary and repeat screening. Results in (**a**, **b**) were from the primary screening and are shown as mean ± standard deviation of five biological replicates. Data from the repeat screening are shown as mean ± standard deviation of three biological replicates. In the scatter plots, each dot refers to one candidate and the red dot indicates the benchmark CD63. The degree of correlation was analyzed with linear regression and is shown as goodness-of-fit (*R*^2^) and significance of non-zero slope (P). Source data are provided as a Source Data file.

Screening the 244 candidates in HEK-293T cells revealed no obvious correlation between intravesicular Tluc and cellular or total secreted Tluc (Supplementary Fig. 1a, b), indicating that neither cellular expression nor overall secretion fully predicts EV-sorting ability. For most candidates, the percentage of intravesicular Tluc was below zero, which is unexpected but might be attributable to attenuated enzyme activity and/or photon lifetime in the presence of the detergent Triton. Nevertheless, it provided a reasonable and practical cut-off for proteins with EV-sorting ability. According to this definition, a total of 36 proteins were found to exhibit EV-sorting ability in HEK-293T cells (Fig. 2a). Among these were five known EV-sorting proteins including three EV markers (CD9, CD63, CD81), one recently identified protein (PTGFRN)^32^, and the viral glycoprotein gag, thereby substantiating the validity of our screening protocol. To the best of our knowledge, this is the first time the remaining 31 proteins have been reported as being capable of luminal cargo loading into EVs.

Out of the four known non-viral EV-sorting proteins, CD63 showed the highest percentage of intravesicular Tluc, with 89% of total secreted Tluc localized inside EVs. Notably, TSPAN2 outperformed CD63 not only in terms of relative intravesicular Tluc (93% vs. 89%) but also absolute amount (2.0e6 vs. 1.7e6; see Source Data file for detailed information). Apart from that, three calmodulin proteins (CALM1, CALM2, CALM3) sorted considerable amounts of Tluc into EVs but with moderate percentages of intravesicular Tluc (29–31%).

To rule out that any unwanted factors interfered with Tluc secretion, suitable quality controls were put in place. First, all candidates were ranked according to total secreted Tluc and the ratio of total secreted Tluc to Nluc. Normalization against Nluc signal did not affect Tluc secretion (Fig. 2b), thereby dismissing a confounding role of the transfection procedure. Secondly, a repeat of the screening revealed consistent results for the percentage of intravesicular Tluc (Fig. 2c) as well as the ranks of total secreted Tluc and secretion efficiency (Fig. 2d). Finally, the ranks of total secreted Tluc and secretion efficiency showed a high degree of linear correlation between two different plasmid doses (0.75 µg/mL vs 1 µg/mL; Supplementary Fig. 1c). Taken together, these results substantiate the reliability of the findings obtained with our screening method.

### EV-sorting proteins are largely conserved across different cell types

Besides HEK-293T, other cell types are regularly used as EV sources prompting us to screen the EV-sorting ability of our candidates in (1) suspension-adapted HEK cells (Freestyle 293-F), (2) human cord blood-derived mesenchymal stem cells (MSCs), (3) human hepatocyte-derived carcinoma cells (Huh-7), and (4) mouse kidney epithelial cells (TCMK-1). For this purpose, 95 candidates that had shown promise in the initial screening, in either of these categories: percentage of intravesicular Tluc, total secreted Tluc, and secretion efficiency (see Source Data file for detailed information), were screened as above.

**Fig. 3:**
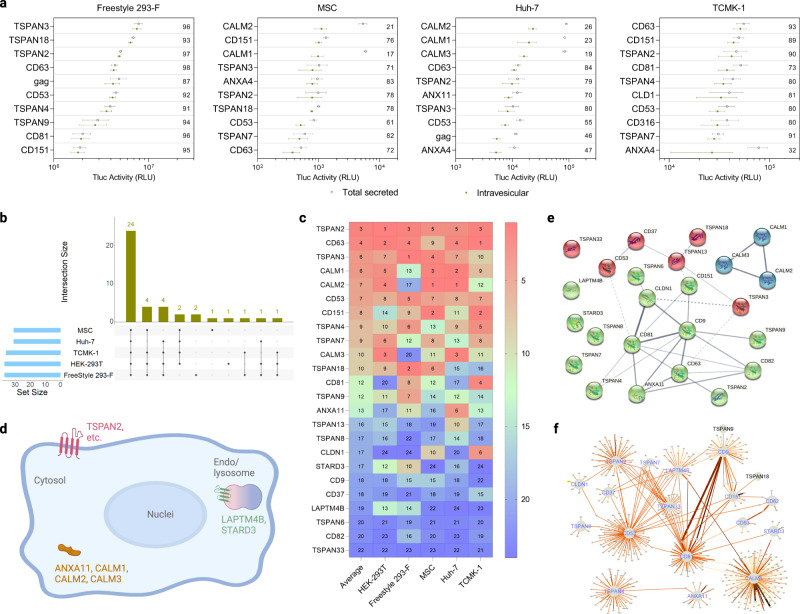
EV-sorting ability of candidate proteins in various cell sources. **a** Top ten scaffold proteins regarding intravesicular Tluc in different producer cell types. The value inside the plot refers to the percentage of intravesicular Tluc. Results are shown as mean ± standard deviation of three biological replicates. **b** Number of EV-sorting proteins identified for each producer cell type and overlap between cell types. **c** Rank of the 24 conserved EV-sorting proteins regarding intravesicular Tluc in each cell type. The value indicates the rank in each cell type as well as the average thereof. **d** Topology and subcellular location of the 24 conserved EV-sorting proteins. Created with BioRender.com. (e-f) Interaction network of the 24 conserved EV-sorting proteins retrieved from STRING (**e**) and IntAct (**f**) databases. Line thickness in panels (**e**, **f**) indicates the strength of data support, with thicker line standing for stronger evidence. Proteins are marked with gene names. Source data are provided as a Source Data file.

In Freestyle 293-F, the protein with the highest EV-sorting ability in terms of percentage of intravesicular Tluc was TSPAN3. In MSCs and Huh-7, CALM2 occupied the highest rank, and in TCMK-1 was CD63 (Fig. 3a). While the transfection procedure was not found to substantially affect Tluc secretion in all the adherent cells tested, Freestyle 293-F seemed more prone to variation (Supplementary Fig. 2). Between 30 and 37 proteins with EV-sorting ability (percentage of intravesicular Tluc above zero) were identified for each cell type (Fig. 3b). Out of these, 24 proteins were conserved across all five cell types indicating their robust sorting ability in different cellular contexts (see Source Data file for a complete list). The proteins in the conserved subset were ranked according to their absolute intravesicular Tluc activity (Fig. 3c). On average, TSPAN2, CD63 and TSPAN3 demonstrated the best sorting abilities across different cell types.

To obtain a more in-depth understanding of potential mechanisms that govern the EV-sorting ability of the conserved subset, bioinformatic studies were conducted. According to the annotation available on UniProtKB, only the three calmodulin proteins and ANXA11 are cytosolic, while the remaining proteins are all members of the tetraspanin superfamily and located either on the plasma or endosomes/lysosomes membrane (Fig. 3d). Next, we evaluated possible interactions between the 24 EV-sorting proteins. The experimental and predicted interactome available from the STRING database showed weak evidence for the interaction of TSPAN2/TSPAN3 with CD63, and calmodulin proteins seem to operate irrespective of the rest (Fig. 3e). Similarly, the results from the IntAct database, which includes both direct and indirect interactions, suggested that the interactome of TSPAN2 overlaps poorly with that of the three well-characterized tetraspanins CD9/CD63/CD81 (Fig. 3f). These predictions indicate that TSPAN2 and TSPAN3 operate largely independent of each other and other tetraspanins.

### EV-sorting candidates prove robust amid standardized EV production

**Fig. 4:**
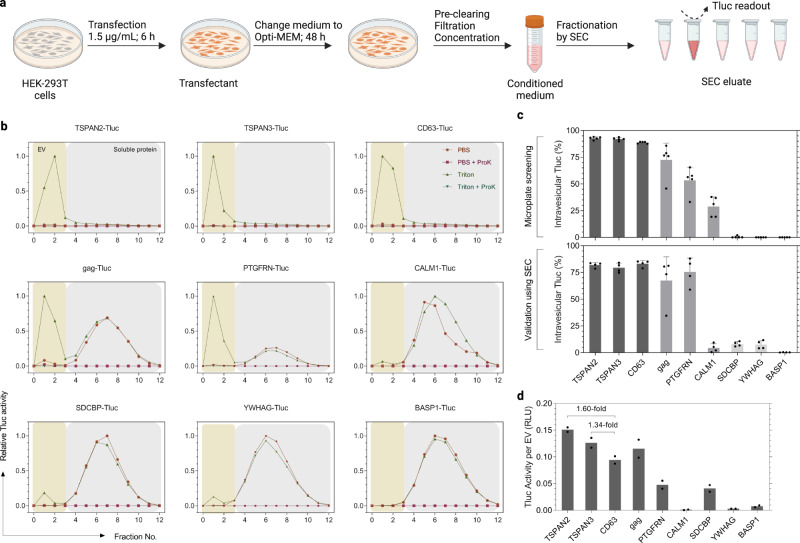
EV-sorting ability of candidate proteins in standardized production conditions. **a** Workflow of EV production and SEC fractionation protocol. Created with BioRender.com. **b** SEC elution profiles of conditioned media from transfected HEK-293T cells. EVs and soluble proteins were recovered in fractions 0–3 and 4–12, respectively. Tluc activity in each fraction was measured with and without Triton and ProK, and normalized to the fraction with the highest signal. **c** Percentage of intravesicular Tluc using the screening (upper panel; five biological replicates) and standardized (lower panel; four biological replicates) protocols. Mean ± standard deviation. **d** Calculated Tluc activity per vesicle for purified EV preparations. Results are shown as an average of two biological replicates. Proteins are marked with gene names. Source data are provided as a Source Data file. SEC size exclusion chromatography.

In the screening, HEK-293T cells were grown and transfected in 96-well microplates for the purpose of higher throughput. Such a scale, however, is not practicable for future applications seeking to produce larger quantities of engineered EVs. Additionally, the conditioned media in the screening were analyzed directly after centrifugation without any defined separation techniques. Here, we were particularly interested in small EVs (sEVs, ≤ 200 nm) because of their therapeutic potential in many diseases^36,37^. With that in mind, we produced EVs according to a standardized protocol recently established by our group^38^. The main differences to the initial screening protocol were (1) a higher dose of plasmids and shorter transfection duration, (2) subsequent maintenance in Opti-MEM, and (3) filtration of conditioned media through a 200-nm membrane followed by a concentration step (Fig. 4a). Additionally, sEVs were separated from soluble proteins by SEC before measuring Tluc activity and vesicle counts (Fig. 4b). Noteworthily, Tluc in the eluate was completely deactivated by Proteinase K indicating that resistant protein aggregates were not a matter of concern (Fig. 4b).

To gain insight into sEV-sorting ability, nine representative candidates were selected based on their performance in the screening (Fig. 4c). The percentage of intravesicular Tluc generally coincided with the screening, showing high (>80%; TSPAN2, TSPAN3, and CD63) and low (<15%; SDCBP, YWHAG, BASP1) sEV-sorting ability (Fig. 4c). Interestingly, CALM1 sorted only 3.7% Tluc into sEVs as compared to 28.9% in the screening (*P* < 0.01, Two-sided Student’s *t* test). Taking vesicle numbers into account, we observed that TSPAN2 and TSPAN3 outperformed CD63 in terms of Tluc activity per EV (1.60-fold and 1.34-fold, respectively; Fig. 4d). Overall, these results demonstrate that the sorting ability of these nine candidates remained largely unchanged when following a standardized sEV production protocol.

### EV-sorting candidates prove versatile for different cargos

**Fig. 5:**
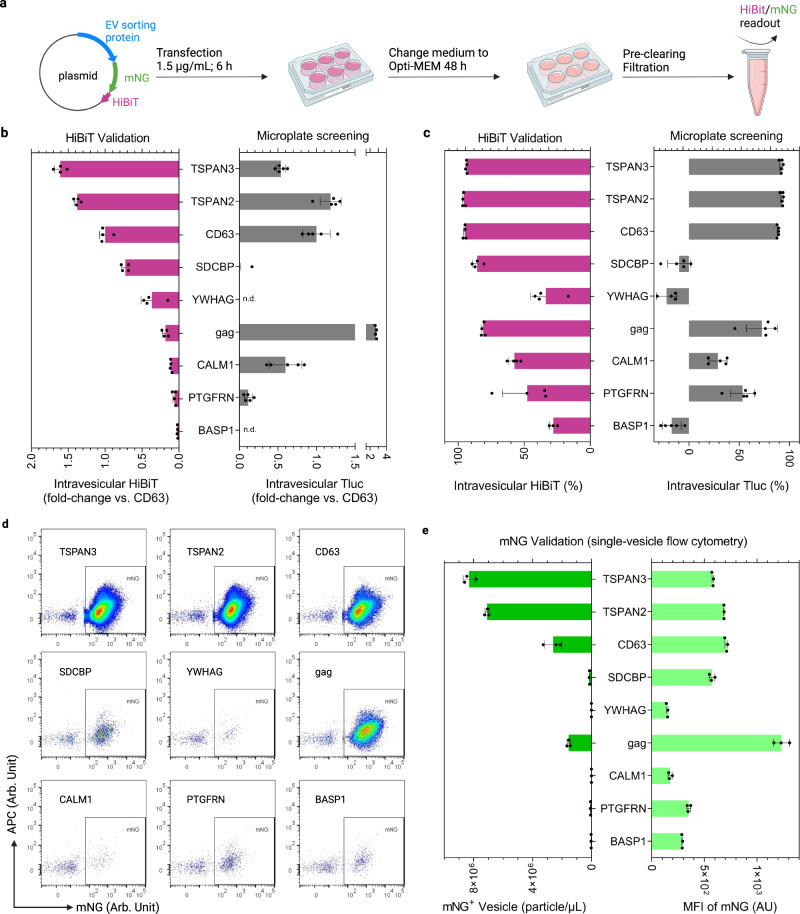
EV-sorting ability of candidate proteins fused to a hybrid bioluminescent and fluorescent reporter. **a** Workflow of EV production and analysis. Created with BioRender.com. **b** Intravesicular HiBiT relative to the benchmark CD63. n.d. not detected. **c** Percentage of intravesicular HiBiT. In **b**, **c**, results are shown as mean ± standard deviation of four biological replicates. Screening results from HEK-293T cells were re-graphed for reference. **d** Single-vesicle flow cytometry dot plots of mNG-HiBiT-labeled EVs. **e** Concentration and mean fluorescence intensity (MFI) of mNG-positive EVs. Results are shown as mean ± standard deviation of three biological replicates. Proteins are marked with gene names. Source data are provided as a Source Data file.

Luciferase is a facile reporter for quantifying engineered EVs in bulk. However, to obtain more information on the number of engineered EVs and the abundance of cargo proteins per EV, single-vesicle imaging flow cytometry is the method of choice^39,40^. Therefore, for the nine candidate proteins explored above, Tluc was replaced with a hybrid reporter consisting of the fluorescent protein mNeonGreen (mNG; 26.6 kDa) fused to HiBiT^41^, an 11-mer peptide from split Nluc luciferase (Fig. 5a).

Comparison of intravesicular luciferase activities of HiBiT and Tluc revealed comparable engineering efficiencies for TSPAN3, TSPAN2 and CD63 in terms of amount (Fig. 5b) and percentage (Fig. 5c). Using mNG to look at the single-vesicle level, TSPAN3 and TSPAN2 produced the highest number of engineered EVs, outperforming CD63 by approximately threefold while reaching similar levels of mNG per engineered EV (Fig. 5d, e). Additionally, we showed that mNG co-localized with respective sorting proteins on the EVs after antibody staining, which is indicative of intact fusion proteins (Supplementary Fig. 3). Surprisingly, HiBiT- and Tluc-based measurements differed greatly for gag (Fig. 5b, c); however, its mNG levels did not show such a discrepancy (Fig. 5d, e). This led us to postulate that the steric configuration of gag-mNG-HiBiT prohibits HiBiT from complexing with its partner subunit to form functional luciferase. Additionally, CALM1 showed low levels of vesicular HiBiT and mNG, in line with the trend observed for CALM1-Tluc in SEC validation experiments (Fig. 4c). These results suggest that CALM1 preferentially sorts into larger vesicles (>200 nm) that are removed during the filtration step (Supplementary Fig. 4).

We additionally evaluated the performance of these selected proteins in Freestyle 293-F cells, which are a prominent source for EV production due to a less tedious propagation procedure. Again, TSPAN3 and TSPAN2 outperformed CD63 by 82% and 50%, respectively, in terms of intravesicular HiBiT (Supplementary Fig. 5a). On a single-vesicle level, the highest concentration of mNG-positive vesicles was produced by TSPAN3 and TSPAN2 engineered Freestyle 293-F cells (Supplementary Fig. 5b). Collectively, the reconciling results based on mNG-HiBiT and Tluc reporters reinforce the reliability of the screening protocol and highlight the robust EV-sorting ability of the candidate proteins for different cargos.

### Distinct molecular signatures among tetraspanin-engineered EVs

Throughout all experiments, TSPAN2 and TSPAN3 appeared among the best EV-sorting proteins, seemingly performing better than the well-characterized tetraspanin CD63. Notably, different splice isoforms of TSPAN2 and TSPAN3 failed to retain EV-sorting ability in HEK293-T cells (Supplementary Fig. 6). Since, as far as we know, this is the first report of these proteins for endogenous engineering of EVs, we characterized the physiochemical features of TSPAN2- and TSPAN3-engineered EVs in relation to CD63-engineered EVs in greater detail.

**Fig. 6:**
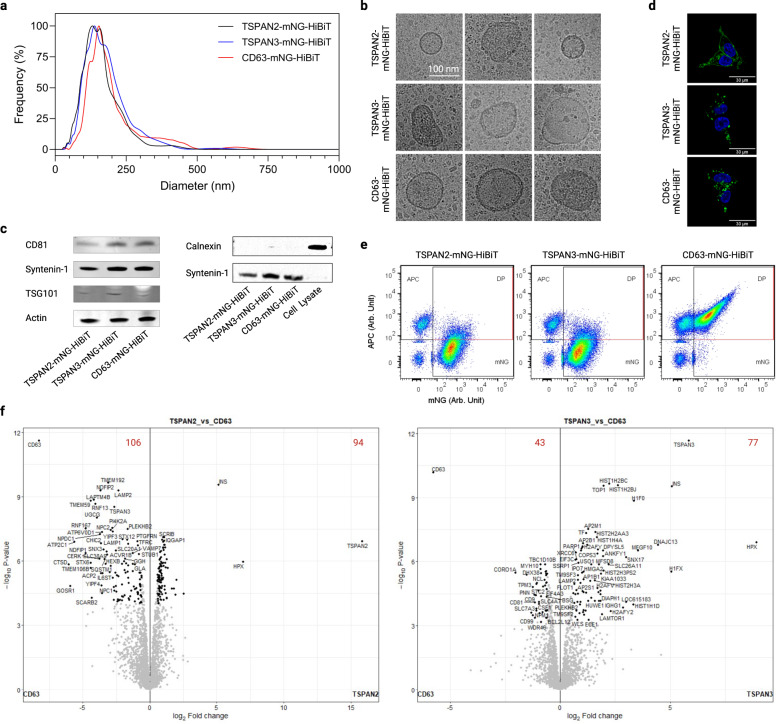
Physiochemical features of TSPAN2-, TSPAN3- and CD63-enriched EVs. **a** Size distribution of EVs from transfected HEK-293T cells. **b** Representative cryo-electron microscopy images of EVs. **c** Western blots of positive and negative markers of EVs. **d** Cellular location of tetraspanins in transfected HEK-293T cells. **e** Single-vesicle flow cytometry dot plots of EVs after staining with APC-conjugated CD9/CD63/CD81 tetraspanin antibodies. **f** Volcano plots showing differentially enriched proteins in EVs. The red digits indicate the count of differentially enriched proteins. Results were from three biological replicates. Source data are provided as a Source Data file. The mass spectrometry proteomics data have been deposited to the ProteomeXchange Consortium via the PRIDE partner repository with the dataset identifier PXD043840.

Nanoparticle tracking analysis of EV preparations from transfected HEK-293T cells revealed a narrow size distribution with median hydrodynamic diameters of approximately 120 nm (Fig. 6a). Also, their morphological appearances were typical of EVs as exemplified by membrane structure and size (Fig. 6b). Moreover, common EV markers such as CD81, syntenin-1 and TSG101, but not the negative marker Calnexin, were detected in the EVs (Fig. 6c). Apart from that, we examined the location of the three tetraspanin proteins in transfected cells to gain insight into EV biogenesis. While TSPAN2 was localized in both the plasma membrane and cytosol of producer cells, TSPAN3 and CD63 were primarily detected as punctate signals inside cells (Fig. 6d). Furthermore, the production of all types of engineered EVs was resistant to inhibition of ceramide (Supplementary Fig. 7a), which is a driver of a recognized exosome production pathway^1,42^.

To get an understanding of their protein signatures, EVs were stained with the classic pan-surface markers CD9/CD63/CD81 and analyzed on a single-vesicle level. Overexpression of the tetraspanins differently affected the yield of total EVs (defined as all fluorescent events, Supplementary Fig. 7b) and engineered EVs (defined as mNG^+^ events, Supplementary Fig. 7c). More interestingly, only a small fraction of TSPAN2- and TSPAN3-engineered EVs displayed the three classic EV markers on surface (Fig. 6e). In addition, EV surface expression analysis of 39 proteins by multiplex bead-based flow cytometry^43^ revealed that the surface epitope composition of TSPAN2-positive EVs differed from that of CD9/CD63/CD81-positive EVs (Supplementary Fig. 7d). Besides surface proteins, in-depth proteomic analyses of EVs showed a plethora of differentially enriched proteins for TSPAN2 (106 de-enriched and 94 enriched) and TSPAN3 (43 de-enriched and 77 enriched) compared to CD63-engineered EVs. CD9/CD63/CD81 were among the proteins that were significantly downregulated in TSPAN2/TSPAN3-engineered EVs, which is in line with the results obtained from single-vesicle and bead-based flow cytometry. Based on Gene Ontology analysis, in comparison with wild-type EVs from HEK293T cells, all three types of engineered EVs (CD63, TSPAN2 and TSPAN3) were enriched (>10%) with metabolite interconversion enzymes, protein modifying enzymes, and RNA metabolism proteins, but depleted (>10%) of extracellular matrix proteins (Supplementary Fig. 8). Next, we compared the overall protein composition with a Principal Clustering Analysis tool and observed that engineered EVs were different from wild-type EVs and to a lesser extent also from each other (Supplementary Fig. 7e). Interestingly, in relative to WT EVs, overexpression of TSPAN2 negatively impacted CD63 and TSPAN3 levels, which could indicate a competitive relationship. TSPAN3 overexpression, on the other hand, led to a slight increase in CD63 levels suggesting a positive regulation (Supplementary Fig. 7f). Overall, these findings illustrate that TSPAN2/TSPAN3-based engineering gives rise to EV subpopulations distinct from CD63.

### TSPAN2- and TSPAN3-engineered EVs as delivery modalities

**Fig. 7:**
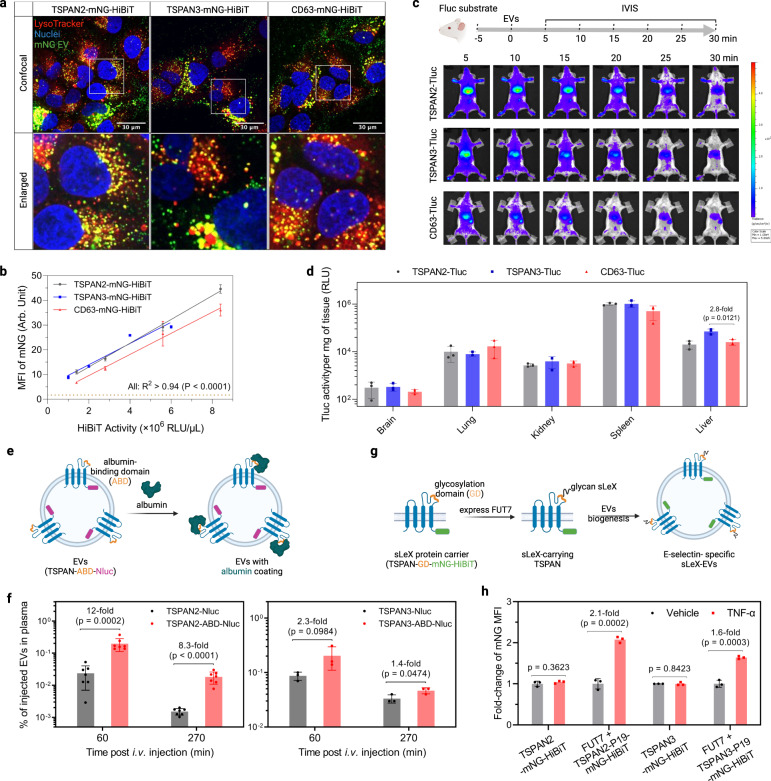
Biological activity of TSPAN2- and TSPAN3-engineered EVs. **a** Huh-7 cells were treated with EVs for 4 h and stained with LysoTracker to visualize lysosomes. Confocal microscopy images from representative regions from the whole well are shown. The experiment was performed once. **b** Huh-7 cells were treated with EVs for 8 h. Cellular mNG MFI was quantified using flow cytometry. Data are shown as mean ± standard deviation of three biological replicates. The degree of correlation was analyzed with linear regression and is shown as goodness-of-fit (*R*^2^) and significance of non-zero slope (P). **c** Biodistribution of EVs in mice. NMRI mice were intraperitoneally injected with d-luciferin substrate. Five minutes later, mice were intravenously injected with the same amount of engineered EVs (based on Tluc activity) and imaged with IVIS. Subsequently, major organs were collected for ex vivo bioluminescence measurements. Representative IVIS images are shown. *N* = 3. **d** Tluc activity in organs ex vivo after IVIS. Results are shown as mean ± standard deviation of three mice. **e** Scheme of generating albumin-binding EVs. EVs were collected from HEK-293T cells stably expressing the fusion proteins. **f** Albumin-binding EVs were injected intravenously and their concentration in plasma was determined. Data are shown as mean ± standard deviation. *N* = 7 (for TSPAN2-related) or 3 (for TSPAN3-related). **g** Scheme of sLeX display on EVs. EVs were collected from HEK-293T cells stably expressing the components. **h** HUVEC cells were activated by TNF-α for 2 h and treated with EVs for 6 h. Cellular mNG MFI was quantified using flow cytometry and is shown as fold-change over un-activated cells. **c**, **e**, **g** Created with BioRender.com. Data are shown as mean ± standard deviation of three biological replicates. Two-sided Student’s *t* test. Source data are provided as a Source Data file.

To explore whether TSPAN2/TSPAN3-engineered EVs are suitable for cellular delivery, we investigated their delivery potential in vitro and in vivo. First, Huh-7 cells were treated with mNG-labeled EVs to examine their subcellular location in recipient cells. The strong punctate yellow signal clearly indicated efficient internalization and trafficking to lysosomes (Fig. 7a). Quantification of cellular MFI using flow cytometry revealed slightly better uptake efficiencies for TSPAN2/TSPAN3-engineered EVs compared to CD63-engineered EVs (Fig. 7b). For EV distribution studies in mice, equal amounts of engineered EVs (based on Tluc activity) were administered intravenously and tracked in real time with in vivo imaging system (Supplementary Fig. 9a and Fig. 7c). For all three types of engineered EVs, we observed rapid distribution to liver and spleen within 5 min (Fig. 7c) and a notable decline in whole-body activity over 30 min (Supplementary Fig. 9c, *P* = 0.0006, Kruskal–Wallis test). TSPAN2- and TSPAN3-engineered EVs seemed to confer slightly higher whole-body retention than CD63-engineered EVs (Supplementary Fig. 9b). Results from subsequent ex vivo measurements supported the dominant hepatic and splenic accumulation of engineered EVs (Fig. 7d). Taken together, like CD63-engineered EVs, TSPAN2- and TSPAN3-engineered EVs are efficiently taken up by cells in vitro and in vivo.

Having extensively showcased the loading and delivery capacity of TSPAN2 and TSPAN3 for luminal cargo, we next investigated their potential for EV surface display applications. The large extracellular loops (LELs) of some tetraspanins have already been exploited for such applications and given the topological similarities of tetraspanin proteins, we sought to engineer the LELs of TSPAN2 and TSPAN3. Insertion of an albumin-binding domain (ABD) into the LEL of CD63, CD9, and CD81 has been shown to drastically extend the plasma circulation time of EVs^44^. Using the same strategy, an ABD was cloned into the LEL of TSPAN2 and TSPAN3 with Nluc at the C-terminus for quantification (Fig. 7e). EVs were collected from HEK-293T cells stably expressing TSPAN-ABD-Nluc fusion proteins (Supplementary Fig. 9c) and assessed for their albumin-binding ability. As expected, only ABD-displaying EVs bound to albumin (Supplementary Fig. 9d). Next, these EVs were intravenously injected into mice and EV concentrations in plasma were measured on the basis of Nluc at different timepoints. In comparison to wild-type tetraspanin-engineered EVs, ABD-displaying EVs had significantly higher concentration in plasma, particularly when using TSPAN2 as the scaffold protein (Fig. 7f).

In another example, we aimed to achieve activated endothelial cell-specific targeting through surface display of the glycan ligand sialyl Lewis X (sLeX)^45^. Therefore, a 19-mer sLeX peptide carrier (P19) was inserted into the LEL of each tetraspanin protein with mNG-HiBiT at the C-terminus. In the presence of fucosyltransferase VII (FUT7), P19 is glycosylated to display sLeX (Fig. 7g). Based on this rationale, sLeX-EVs were produced from HEK-293T cells stably expressing FUT7 and TSPAN-P19-mNG-HiBiT (Supplementary Fig. 9c). Their uptake was evaluated in TNF-α-activated endothelial cells, which express E-selectin, the main receptor for sLeX. Wild-type tetraspanin-engineered EVs were taken up similarly in un-activated and activated endothelial cells while sLeX-EVs, using either TSPAN2 or TSPAN3 as the scaffold, demonstrated preferable uptake by activated endothelial cells (Fig. 7h). Overall, this demonstrates the feasibility of TSPAN2 and TSPAN3 in terms of simultaneous engineering of the LEL for surface display and C-terminus for luminal cargo loading, thus highlighting their potential for therapeutic applications.

## Discussion

In this study, we successfully established a simple and robust assay to quantify intravesicular protein cargoes, which is fundamental to compare EV-sorting ability of candidate proteins in a large scale. In doing so, we identified TSPAN2 and TSPAN3 as highly efficient EV-sorting candidates. Both TSPAN2 and TSPAN3 consistently appeared among the top-performing candidates across different cell lines and for different cargo proteins (sizes ranging from 26.6 kDa to 60.5 kDa). Furthermore, the cellular uptake of TSPAN2- and TSPAN3-engineered EVs in vitro and in vivo demonstrates their potential as improved delivery modalities. The finding that neither TSPAN2 nor TSPAN3 are highly expressed in EVs from wild-type HEK-293T cells (Supplementary Fig. 7d) might explain why they had been neglected in previous EV-engineering studies. Consequently, little is known about their functions, especially in relation to EVs. TSPAN2 is hypothesized to be involved in oligodendrocyte differentiation and cancer metastasis^46^. Less is known about the functions of TSPAN3 besides a link to the progression of acute myeloid leukemia^47^, however, its presence in urinary exosomes has been shown^48^. Nevertheless, our functional characterization of TSPAN2- and TSPAN3- engineered EVs suggests similar internalization dynamics and fate as CD63 in vitro and in vivo.

The majority of candidates (21 out of 24) with conserved EV-sorting ability across different cell types was found to belong to the tetraspanin superfamily, demonstrating the importance of this topological feature for EV sorting. While tetraspanins are implicated in a variety of cellular processes, some of them have been found to aid membrane curvature therefore implying a role in EV biogenesis^49,50^. By inhibiting ceramide-dependent EV biogenesis, we showed that none of TSPAN2-, TSPAN3- or CD63-engineered EVs are produced via this route. Other studies suggest an ESCRT-independent pathway for CD63^51,52^, which altogether supports the concept of a tetraspanin-specific route of EV biogenesis^53^. Consequently, overexpression of tetraspanins could boost the production of (engineered) EVs. However, some family members might be more useful to that end than others, which is reflected in their heterogenous EV-engineering ability observed here. This is also connected to our and others’ observations that distinct tetraspanin subpopulations of EVs exist^27,54^. Hence, we hypothesize that overexpression of tetraspanins results in the production of certain EV subpopulations, thereby dictating its EV-engineering potential. However, tetraspanins seem unlikely to contribute to cellular uptake in recipient cells. Unraveling the individual implications of tetraspanins in EV biogenesis and targeting is a matter of concern for future studies.

Interestingly, we found that calmodulins preferentially sort cargo into larger EVs (>200 nm). Given that larger EVs are commonly generated by outward budding of the plasma membrane, we believe that calmodulins are predominantly present in microvesicles (100–1000 nm). In support of that, CALM1 showed weak putative interactions with typical markers of small EVs produced in the endosomal system, i.e., exosomes. This finding can be particularly useful to applications involving large EVs for drug or gene delivery.

Although we aimed to address various aspects of endogenous EV engineering, future studies will provide further insight into the EV-sorting ability of our candidates in other settings. Cargo proteins here were fused to the C termini of all 244 proteins, leaving no interpretation for other fusion sites. Although our screening was initially designed to compare luminal cargo proteins of different EV-sorting candidates, we showed that the lead proteins TSPAN2 and TSPAN3 were tolerable to simultaneous surface engineering and luminal cargo loading. Additionally, it remains to be determined whether the EV-sorting ability observed here upholds for alternative cargo molecules. These or other reasons could potentially explain why we were unable to observe superior EV-sorting ability for BASP1 and PTGFRN as seen in a previous study^32^. Apart from that, it is not surprising to observe producer cell-dependent EV sorting ability. HIV-derived gag, for example, displayed impressive sorting ability in HEK-293T cells but not in MSCs (Fig. 3b, see Source Data file). Other candidates were found to be exclusive to certain cell types likely influenced by the cell’s transcriptome and proteome, therefore demanding individual screenings. On the upside, a conserved subset of proteins was identified that we hypothesize to be involved in core physiological processes of EV production. These candidates lend themselves as reliable EV-engineering candidates irrespective of the producing cell.

In conclusion, this study is by far the most comprehensive report of its kind that examined the EV-sorting potential of overexpressed proteins. Hence, it will provide a valuable reference point for researchers aiming to sort cargo into EVs by endogenous means. Additionally, TSPAN2 and TSPAN3 are identified as reliable and efficient EV-sorting proteins, which poses a steppingstone for endogenous engineering strategies and might in foresight broaden the applications of EVs as delivery modalities.

## Methods

### Ethical statement

All mouse experiments were performed in accordance with the ethical permission granted by Swedish Jordbruksverket (permit No.13849-2020).

### Cloning

Codon-optimized DNA sequences coding for the scaffold protein and luciferase reporter were cloned downstream of the CAG promoter into the pLEX vector (Twist Bioscience, US). ABD and P19 peptides were inserted between amino acid (aa) 154–155 for TSPAN2 and aa 150–151 for TSPAN3, respectively. To generate different constructs expressing mNG-HiBiT or Nluc, protein-coding sequences for Tluc were replaced with corresponding fragments through In-Fusion cloning (Takara; 638948) or restriction cloning strategies. All expression cassettes were confirmed by sequencing. Scaffold protein identifiers are listed in Data Source file. Plasmids are available from the corresponding author upon request.

### Cell culture

HEK-293T (ATCC, CRL-3216), Huh-7 (XenoTech, JCRB0403) and TCMK-1 (ATCC, CCL-139) cells were maintained in high glucose DMEM media (Gibco, 41966-029) supplemented with 10% fetal bovine serum (FBS; Gibco, 10270-106) and 1% anti-anti (Gibco, 15240). HUVEC (ATCC, CRL-4053) cells were cultured in Endothelial Cell Growth Medium MV2 (PromoCell, C-22022) supplemented with 1% anti-anti. Human cord blood-derived MSCs (ATCC, PCS-500-010) were cultured in MEM media (Gibco, 22561-021) supplemented with 10% FBS and 1% anti-anti. Freestyle 293-F (ThermoFisher, R79007) cells were kept in FreeStyle 293 Expression Media (12338-018) under continuous shaking at 175 rpm. All cells were cultured in humidified incubators with 37 °C and 5% CO_2_.

### Transfection

In the screening protocol, producer cells (HEK-293T, Huh-7 and TCMK-1) were seeded in 96-well plates (100 µL media per well) and transfected when approximately 30% confluent. Freestyle 293-F cells were seeded at 7.5 × 10^4^ cells per well (90 µL media per well) and directly transfected. For transfection, 10 µL of plasmid-lipofectamine mixture containing 75 ng plasmid and 165 ng Lipofectamine 2000 (Invitrogen, 11668-019) in Opti-MEM (Gibco, 31987-047) were added to each well. The cells were cultured for an additional 48 hours before the conditioned media were collected.

In six-well plates, HEK-293T cells were seeded in 2 mL media per well and transfected when approximately 60% confluent. Three micrograms of plasmid were complexed with 6.6 µg of Lipofectamine 2000 in 200 µL Opti-MEM and added to each well. The cells were cultured for an additional 48 h before the conditioned media were collected.

In 15-cm petri dishes, HEK-293T were seeded in 20 mL media per petri dish and transfected when approximately 60% confluent. Thirty micrograms of plasmid were complexed with 45 µg of polyethyleneimine (Polysciences; 24765-1) in 4 mL Opti-MEM and added to each dish. Transfection was discontinued 6 h later by changing the media to Opti-MEM and the cell culture was maintained for an additional 48 h before harvesting conditioned media.

### Establishing stable cell lines

Lentivirus encoding transgenes of interest were produced in HEK-293T cells according to our previous reports^45,55^. To generate stable cell lines, HEK-293T cells were cultured in six-well plates until approximately 60% confluent and then transduced with lentiviral particles overnight. Transduced cells were expanded and selected using 4 µg/mL of puromycin (Sigma-Aldrich, P8833).

### Isolation of extracellular vesicles

In the screening protocol, conditioned media from transfected cells was pre-cleared by two rounds of centrifugation (700 × *g* for 5 min and then 2000 × *g* for 10 min) to pellet cells and cell debris. If not specified, the supernatant was filtered through a 200 nm membrane to remove large particles. To obtain enough EVs for size exclusion chromatography (SEC), 20 mL of the processed media was concentrated to approximately 1 mL using Amicon Ultra-2 spin-filter with 10 kDa molecular weight cut-off (Millipore, UFC201024).

To produce EVs in larger scale, we followed a protocol previously reported by our group^38^. Briefly, after pre-clearing and filtration, large volumes of conditioned media were diafiltrated and concentrated to roughly 50 mL using the KrosFlo KR2i TFF System (Repligen, US) with 300 kDa cut-off hollow fiber filters (Spectrum Labs, D06-E300-05-N) at a flow rate of 100 mL/min (transmembrane pressure at 3.0 psi and shear rate at 3700 sec^−1^)^56^. EVs were further concentrated until approximately 500 μL using Amicon Ultra-15 spin-filter with 100 kDa molecular weight cut-off (Millipore, UFC910024) and stored at −80 °C in PBS-HAT buffer^57^ before downstream analysis.

EVs for proteomics study were purified using the automated chromatography system (GE Healthcare Life Sciences, ÄKTA Start). Briefly, EVs were separated on a HiTrap Capto Core 700 column (GE Healthcare Life Sciences) and collected according to the absorbance from a 280 nm UV detector. Samples were further concentrated using 10 kDa cut-off spin-filters (Millipore, UFC901024) and stored at −80 °C before use.

### Size exclusion chromatography

Five hundred microliters of concentrated conditioned media was loaded onto SEC columns (Izon, SP1). After discarding the void fraction (first 2 mL), a total of 13 fractions of the eluate (1 mL per fraction) was collected sequentially and numbered as 0–12. According to the manufacturer’s instruction, fraction 1–3 were enriched with EVs and pooled as the EV fraction for downstream analysis. For better separation, the eluate was collected into 48 fractions (0.3 mL per fraction). To check albumin-binding ability in vitro, EVs were incubated with FITC-HSA (Abcam, ab8030) at 37 °C for 2 h before SEC separation.

### Nanoparticle tracking analysis

Particle size and concentration were measured via nanoparticle tracking analysis (NTA) using NanoSight NS500 equipped with NTA 3.2 analytical software (Malvern Panalytic, UK). Briefly, samples were diluted in 200-nm-filtered PBS if required and acquired using the following settings: five 30-s videos were recorded per sample with a camera level of 13. Software settings for analysis were kept constant for every measurement (screen gain 20, minimum track length 3).

### Western blotting

EVs (2 × 10^9^ in 24 µL) were mixed with 8 µL of sample buffer (0.5 M dithiothreitol, 0.4 M sodium carbonate, 8% sodium dodecyl sulfate and 10% glycerol) and heated at 70 °C for 10 min. The mixture was loaded onto a NuPAGE Novex 4–12% Bis-Tris Protein Gel (Invitrogen, NP0335BOX) and separated at 120 V in NuPAGE MES SDS running buffer (Invitrogen, NP0002) for 2 h. Proteins on the gel were transferred to an iBlot nitrocellulose membrane (Invitrogen, IB23001) for 7 min using the iBlot system. Membranes were blocked with Odyssey blocking buffer (LI-COR, 927-60004) for 1 h under gentle shaking. Afterwards, the membrane was incubated overnight at 4 °C with primary antibody solution (1:1000 dilution for anti-TSG101 [Abcam, ab30871], anti-Calnexin [ThermoFisher, PA5-19169] and anti-Syntenin-1 [Origene, TA504796]; 1:200 dilution for anti-CD81 [SantaCruz, sc-9158] and 1:10,000 dilution for anti-β-actin [Sigma, A5441]). The membrane was rinsed with PBS supplemented with 0.1% Tween 20 (PBS-T) for 3 times over 15 min and incubated with the corresponding secondary antibody (925–68070, 926–68071, 926–32210, 926–32211; 1:15,000 dilution for all, LI-COR) for 1 h. Membranes were rinsed with PBS-T for 3 times over 15 min, one time with PBS and visualized on the Odyssey infrared imaging system (LI-COR, US).

### Proteomics analysis

EVs produced by wild-type (WT) or transduced stable cell lines were subjected to proteomic analysis. Briefly, samples were run with ThermoFisher Scientific Q Exactive Plus LC-MS/MS with 1 h gradient and analyzed using R (version 4.1.2, 2021-11-01), RStudio (version 2022.07.1+554) and DEP (version 1.16.0). After filtering out bovine serum-derived proteins, keratin, mNG and puromycin resistance proteins, a total of 2340 proteins across all samples were included for downstream analysis. All differentially enriched proteins were also subjected to Gene Ontology analysis and classified into Protein Ontology classes using PANTHER 17.0 software. The mass spectrometry proteomics data have been deposited to the ProteomeXchange Consortium via the PRIDE partner repository with the dataset identifier PXD043840.

### Cryo-electron microscopy

Four microliters of sample was adsorbed onto holey carbon-coated grid (Quantifoil, Germany) that was glow-discharged. After blotting with filter paper, the grid was vitrified into liquid ethane at −178 °C using a Vitrobot (FEI, Netherlands). The frozen grid was then transferred onto a Philips CM200-FEG electron microscope (FEI, Netherlands) using a Gatan 626 cryo-holder (GATAN Inc, USA). Electron images were acquired using a low-dose system (accelerating voltage of 200 kV; nominal magnification of 50,000; temperature of −175 °C). Defocus values ranged from −2 µm to −3 µm. Micrographs were recorded using a CMOS camera (TVIPS, Germany) at 4 K × 4 K.

### Confocal microscopy

Huh-7 cells were seeded at 10,000 per well in glass-bottom chamber slides (ThermoFisher; 155409) and incubated overnight. The cells were treated with HiBiT-mNG-labeled EVs (1 × 10^7^/µL) and incubated for 3.5 h. Hoechst 33342 (ThermoFisher, H3570) and LysoTracker Red DND-99 (ThermoFisher, L7528) was added to visualize nuclei and lysosomes, respectively. At 4 h, chamber slides were transferred to a microscope stage-top incubator at 37 °C with 5% CO_2_. Imaging was conducted using a confocal microscope (A1R confocal, Nikon, Japan) and analyzed by the NIS-Elements software (Nikon, Japan). 3D reconstruction was compiled with a height of 10 μm and a resolution of 0.3 μm.

### Flow cytometry for cells

Huh-7 cells were seeded at 3 × 10^4^ cells per well in 96-well plate and incubated overnight. The cells were treated with mNG-HiBiT-labeled EVs for 8 h. HUVECs were seeded at 5 × 10^4^ cells per well in 24-well plates and incubated overnight. The cells were stimulated with TNF-a (20 ng/mL) for 2 h and treated with mNG-HiBiT-labeled EVs for another 6 h. After trypsinization, cells were resuspended in 100 µL of PBS containing 2% FBS. 4ʹ,6-diamidino-2-phenylindole (DAPI) was added to all samples to exclude dead cells. The samples were measured with MACSQuant Analyzer 10 cytometer (Miltenyi, Germany). Data was analyzed with FlowJo software (version 10.6.2) and doublets were excluded by forward scatter area versus height gating (gating strategy available in Supplementary Fig. 10).

### Flow cytometry for multiplex beads

MACSPlex Exosome Kit (Miltenyi Biotec; 130-108-813) was used to characterize the surface protein composition of EVs following manufacturers’ instructions. In brief, EVs (1 × 10^9^ in 120 µL) were incubated with 15 µL of MACSPlex exosome capture beads overnight in wells of a pre-wet and drained MACSPlex 96-well 0.22 µm filter plate at room temperature. The beads were rinsed with 200 µL MACSPlex buffer and detected after staining with APC-conjugated antibody mixture (anti-CD9/CD63/CD81) or AF647-conjugated anti-TSPAN2 antibodies (FAB7876R; R&D systems) for 1 h at room temperature. Next, the samples were rinsed twice, resuspended and analyzed using MACSQuant Analyzer 10 flow cytometer. FlowJo (v.10.6.2) was used to analyze data. Median fluorescence intensities (MFIs) for all 39 capture bead subsets were background-corrected by subtracting the respective values from matched non-EV-containing buffer controls and normalized to the beads with the highest level. Gating strategy was the same as in our previous report^43^.

### Flow cytometry for single vesicle

mNG-HiBiT-labeled EVs were analyzed at a single-vesicle level on an Amnis CellStream instrument (Luminex) equipped with 405, 488, 561 and 642 nm lasers based on previously optimized settings and protocols^39^ and as described recently^57^ In brief, EV samples at a concentration of 1 × 10^10^ particles/mL were incubated with AF647-labeled anti-TSPAN2 antibody (R&D systems, FAB7876R-100UG, 0.5 µL) or a mixture of APC-labeled anti-CD9 (Miltenyi Biotech, clone SN4), anti-CD63 (Miltenyi Biotec, clone H5C6) and anti-CD81 antibodies (Beckman Coulter, clone JS64) at a concentration of 8 nM overnight and diluted by 2000-fold in PBS-HAT buffer before data acquisition. Samples were measured with FSC turned off, SSC laser set to 40%, and all other lasers set to 100%. EVs were defined as SSC (low) by using mNG-tagged EVs as biological reference material, and regions to quantify mNG^+^ or AF647/APC^+^ fluorescent events were set according to unstained non-fluorescent samples and single fluorescence positive mNG-tagged reference EV controls. Data were analyzed using FlowJo software (version 10.6.2, gating strategy available in Supplementary Fig. 10).

### Mouse experiments

Female NMRI mice were bought from Charles River and housed in our animal facility for at least one week before use according to standard routines (ambient temperature: 20–22 °C, humidity: 45–55%, dark/light cycle: 12/12 h). To check biodistribution of Tluc-labeled EVs, female NMRI mice with a bodyweight of around 25 g (age around 4–6 weeks) were administered intraperitoneally with 150 mg/kg D-luciferin (PerkinElmer, 122799). After 5 min, EVs (containing the same amount of Tluc in 100 µL) were injected through the tail vein. Live animals (isoflurane sedated) were imaged every 5 min over 30 min by IVIS Spectrum (PerkinElmer, USA) with an exposure time of 30 s. Immediately after completion of IVIS session, the mouse was bled for collecting blood in EDTA-coated tubes and sacrificed for collecting major organs. Blood samples were immediately centrifuged at 2000 × *g* for 10 min to retrieve plasma. The organs were weighed and lysed in 1 mL Triton X-100 solution (0.1% in PBS) using a TissueLyser II (QIAGEN, Germany) according to the manufacturer protocol. To analyze plasma retention of EVs, Nluc-labeled EVs were injected through tail vein. Blood samples were taken 60 min and 270 min after injection to retrieve plasma.

### Luciferase detection assay

Tluc was quantified in five types of samples: cell lysate, conditioned media, SEC eluate, mouse plasma and tissue lysate. To liberate Tluc from cells, the cell pellet was suspended in 100 µL Triton X-100 solution (0.1% in PBS) and shaken horizontally at 500 RPM for 10 min. Mouse plasma and tissue lysate were diluted by fivefold and tenfold with Triton X-100 solution, respectively. Typically, 25 μL of samples was added into white-walled 96-well plates and an equal volume of ready-to-use Tluc substrate (Promega; E1501) was injected to each well. The luciferase intensity in each well was immediately measured using a GloMax 96 Microplate Luminometer machine (Promega, USA). For conditioned media, 25 μL of samples was mixed with 25 μL PBS or Triton X-100 solution. The plate was shaken horizontally at 500 RPM for 5 min before the addition of Tluc substrate. To discern resistant protein aggregate in SEC eluate, 25 µL of samples was mixed with Triton X-100 solution and then incubated with 25 µL of Proteinase K (ProK; Qiagen, 19131; 100 µg/mL in PBS) at 37 °C for 30 min prior to Tluc measurement.

To detect Nluc in conditioned media and plasma, 25 μL of samples was added into white-walled 96-well plates along with 25 µL of Triton X-100 solution. The plate was shaken horizontally at 500 RPM for 5 min before addition of 25 µL of ready-to-use Nano-Glo substrate (Promega; N1130) for measuring luciferase intensity.

To detect HiBiT in conditioned media, 25 μL of samples was added into white-walled 96-well plates along with 25 µL PBS or Triton X-100 solution. The plate was shaken horizontally at 500 RPM for 5 min. Fifty microliters of ready-to-use HiBiT Lytic Detection mixture (Promega; N3040) was added to each well. After incubation at room temperature under horizontal shaking at 500 RPM for 10 min, the plate was immediately measured.

### Statistics and reproducibility

Results were as mean (± standard deviation) of biological replicates if applicable. Two-sided Student’s *t* test was used to compare the difference between two groups. No statistical method was used to predetermine sample size. No data were excluded from the analyses. Mice and samples were randomized into study groups. The Investigators were not blinded to allocation during experiments and outcome assessment.

### Reporting summary

Further information on research design is available in the Nature Portfolio Reporting Summary linked to this article.

### Supplementary information

Supplementary Information

Reporting Summary

### Source data

Source Data

## Data Availability

The mass spectrometry proteomics data have been deposited to the ProteomeXchange Consortium via the PRIDE partner repository with the dataset identifier PXD043840. Source data are provided with this paper.
