# Supplementary material for: Identification of scaffold proteins for improved endogenous engineering of extracellular vesicles
Source: Nat Commun. 2023 Aug 7;14:4734. doi: 10.1038/s41467-023-40453-0 (PMC10406850; doi:10.1038/s41467-023-40453-0)
Supplement: Supplementary file 1 — Supplementary Information [file 41467_2023_40453_MOESM1_ESM.pdf]

Identification of Novel Scaffold Proteins for Improved Endogenous Engineering of Extracellular Vesicles

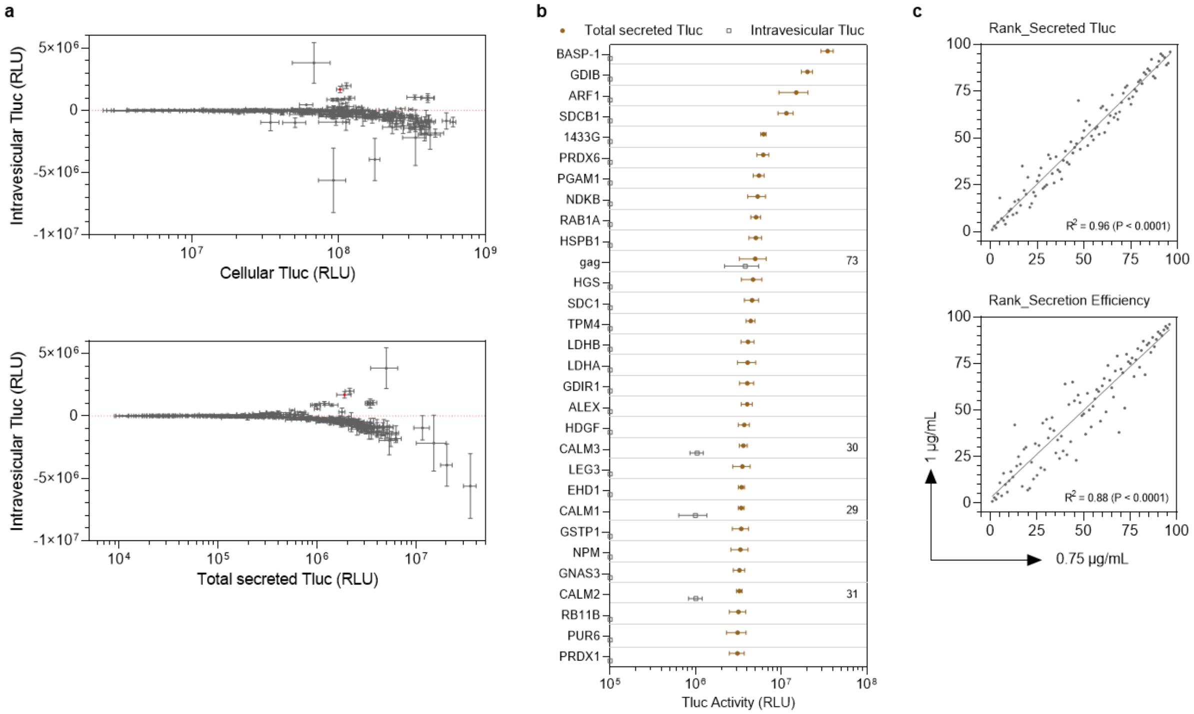

Supplementary Figure 1. Screening results on HEK-293T cells. (a) Overview of intravesicular Tluc in relation to cellular Tluc and total secreted Tluc. (b) Top 30 proteins in terms of total secreted Tluc in the primary screening. Mean ± standard deviation of five biological replicates. Proteins are marked as gene names. The value adjacent to the right axis refers to the percent intravesicular Tluc. (c) Correlation regards the rank of secreted Tluc and secretion efficiency at different plasmid dose. Each dot refers to one candidate protein. Three biological replicates for each protein. The degree of correlation was performed using linear regression and shown as goodness-of-fit (R^2) and significance of none-zero slope (P). Source data are provided as a Source Data file.

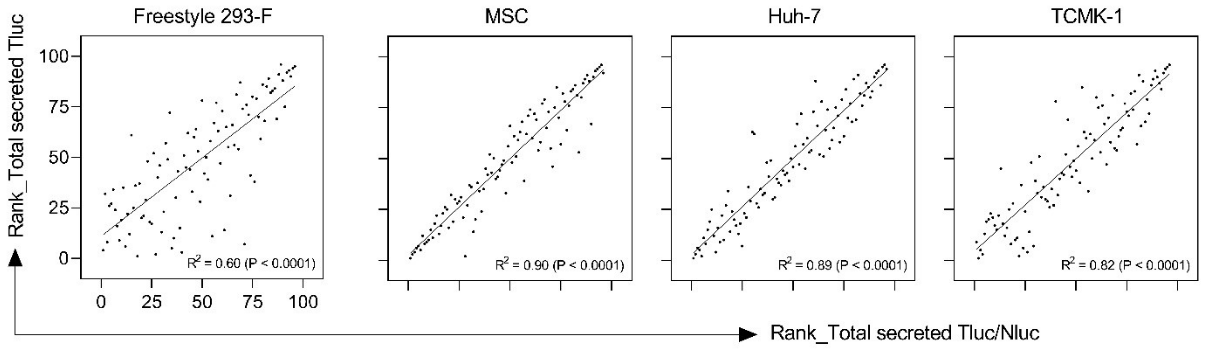

Supplementary Figure 2. Correlation between the rank regarding total secreted Tluc and that regarding total secreted Tluc/Nluc in different producer cell lines. Three biological replicates for each protein. The degree of correlation was performed using linear regression and shown as goodness-of-fit (R^2) and significance of none-zero slope (P). Source data are provided as a Source Data file.

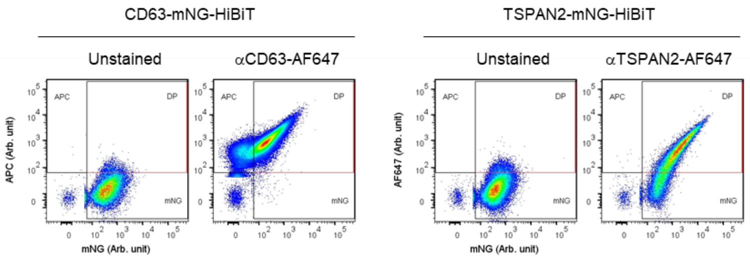

Supplementary Figure 3. Detection of TSPAN2 and CD63 on engineered EVs using single-vesicle imaging flow cytometry. EVs were from transfected HEK-293T cells.

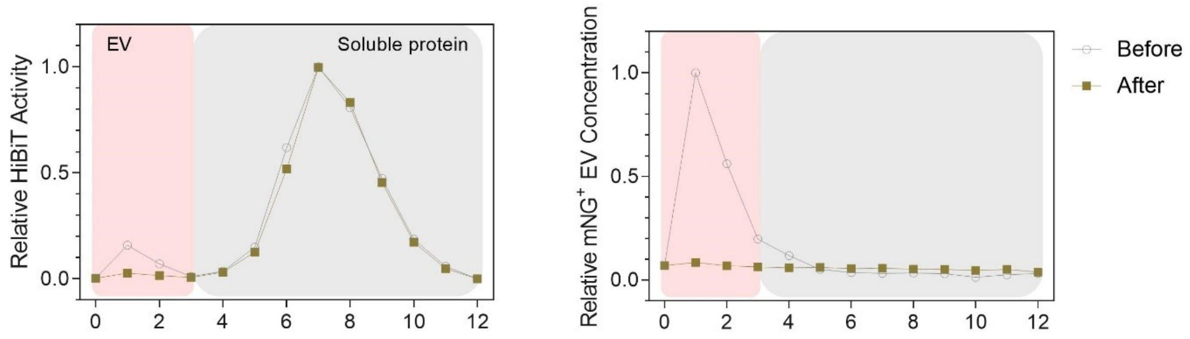

Supplementary Figure 4. Effect of filtration on CALM1-engineered EVs. The conditioned media of CALM-HiBiT-mNG-transfected HEK-293T cells were directly fractionated or fractionated after 0.2 μm filtration. HiBiT activity and mNG^+ EV were

quantified using bioluminescence and flow cytometry, respectively. Data were normalized to the fraction with maximum level. Source data are provided as a Source Data file.

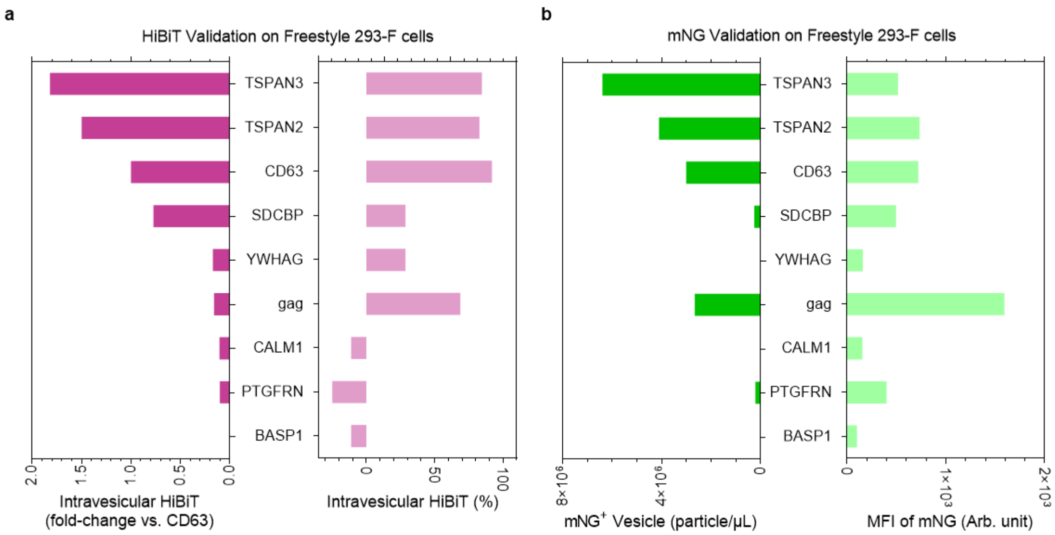

Supplementary Figure 5. Quantification of HiBiT and mNG in the secretome of transfected Freestyle 293-F cells. Freestyle 293-F cells were grown in 6-well plates and transfected with 1.5 μg/mL plasmid for 48 hr. The conditioned media were pre-cleared and filtered through 0.2 μm membrane. (a) Quantification of intravesicular HiBiT and percentage of intravesicular HiBiT. Data was normalized to CD63. (b) Quantification of the concentration and mean fluorescence intensity (MFI) of engineered EVs using single-vesicle flow cytometry. Proteins are marked as gene name. Source data are provided as a Source Data file.

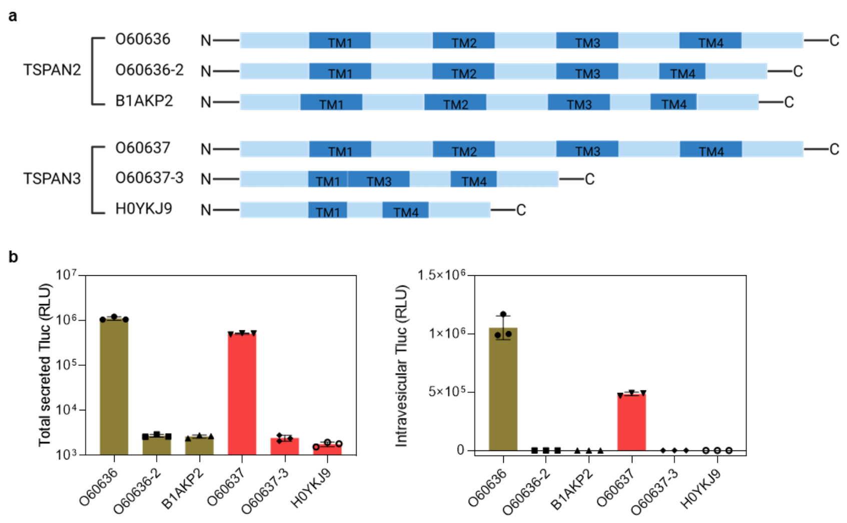

Supplementary Figure 6. EV-sorting ability of TSPAN2 and TSPAN3 isoforms. (a) Topological scheme of isoforms. TM refers to transmembrane submain. Tluc was fused to the C-terminal of the isoforms. (b) HEK-293T cells were grown in 96-well microplates and transfected with 0.75 µg/mL plasmid for 48 hr. After centrifugation, total secreted and intravesicular Tluc in the conditioned medium were quantified. Mean ± standard deviation of three biological replicates. Source data are provided as a Source Data file.

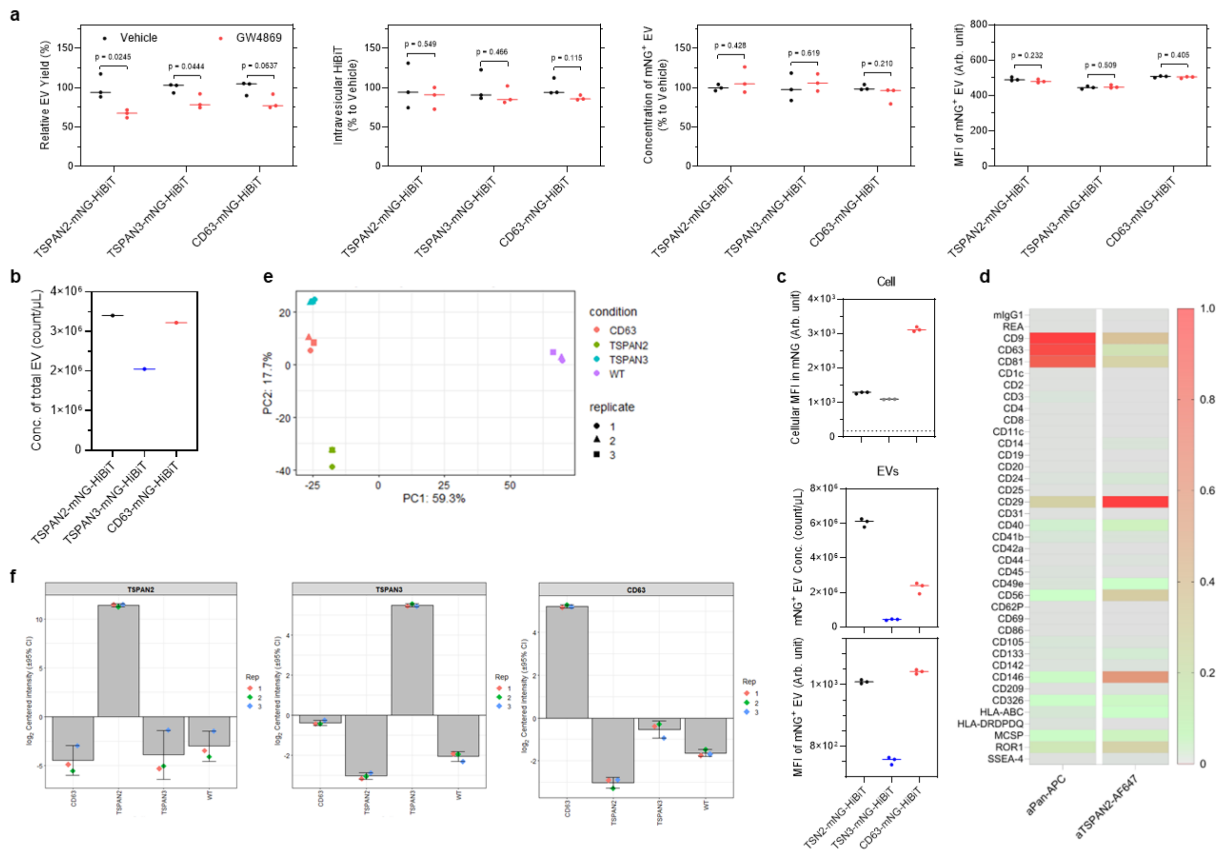

Supplementary Figure 7. Physiochemical characterization of engineered EVs. (a) Effect of GW4869 on the yield of total and engineered EVs from transfected HEK-293T cells. GW4869 was added to Opti-MEM at a final concentration of 5 μm. Results are shown as mean ± standard deviation of three biological replicates. Two-sided Student's *t* test. (b) Quantification of total EVs in Figure 6e. (c) HEK-293T cells stably expressing the transgenes were seeded in 6-well plates at the same cell number for producing EVs. Cells and EVs were analyzed by respective flow cytometers. Mean ± standard deviation of three biological replicates. (d) Surface epitope composition of EVs from TSPAN2-Tluc-transfected HEK -293T cells. EVs were captured with indicated beads (MACSPlex Exosome Kit, human), stained with Pan (CD9/CD63/CD81) or TSPAN2 detection antibodies, and detected by flow cytometry. (e-f) Principal clustering analysis (e) and relative tetraspanin expression (f) of wildtype (WT) and engineered HEK-293T EVs using proteomics dataset. Engineered EVs were all labeled with mNG-HiBiT. Mean ± standard deviation of three biological replicates The mass spectrometry proteomics data have been deposited to the ProteomeXchange Consortium via the PRIDE partner repository with the dataset identifier PXD043840. Other source data are provided as a Source Data file.

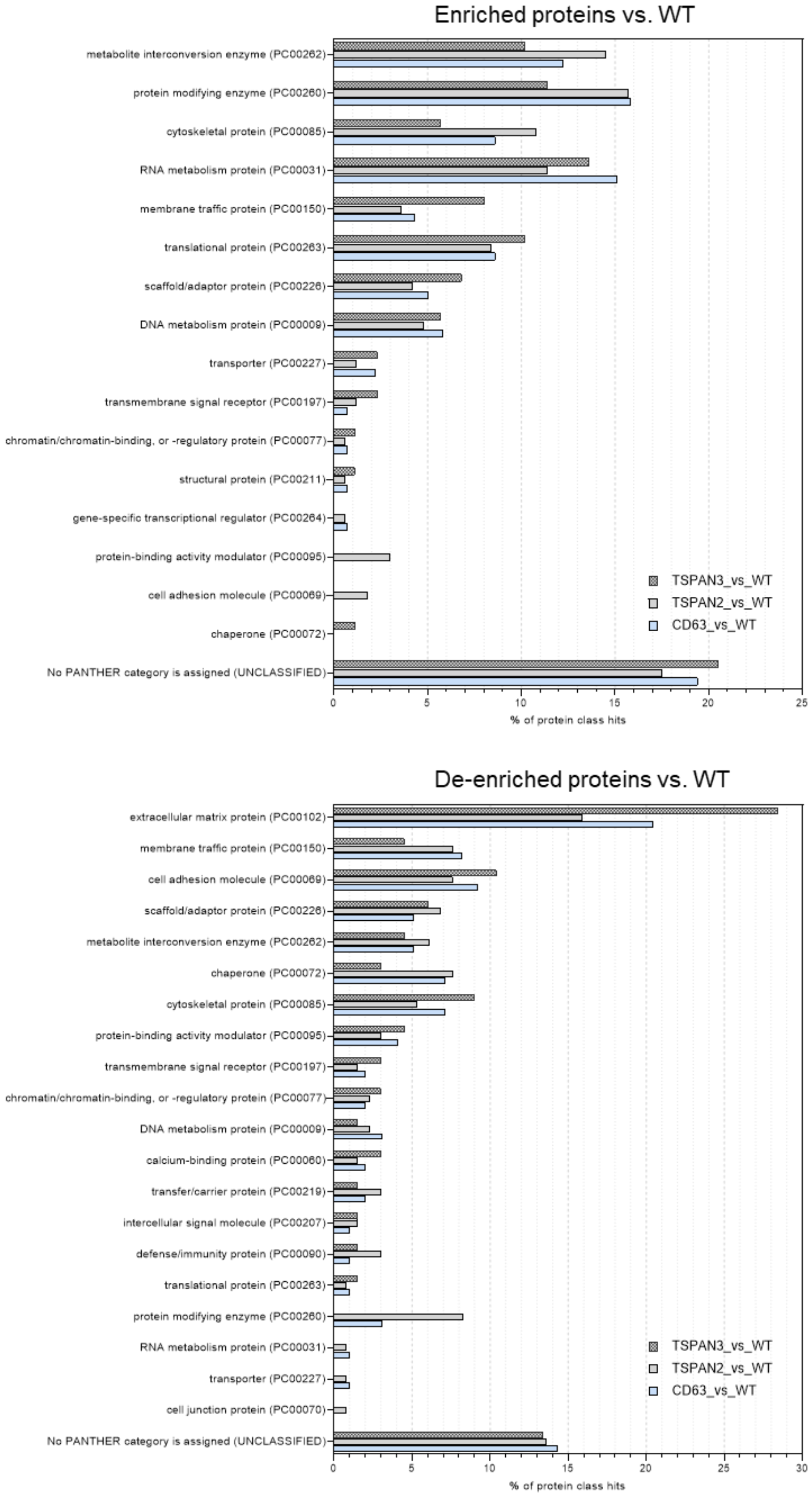

Supplementary Figure 8. Gene ontology analysis of enriched and de-enriched proteins in engineered EVs compared to wildtype EVs. The % of protein class hits indicates to

the percentage of engineered EV differentially enriched/de-enriched proteins that were assigned a Protein Ontology class by PANTHER 17.0 software. The mass spectrometry proteomics data have been deposited to the ProteomeXchange Consortium via the PRIDE partner repository with the dataset identifier PXD043840.

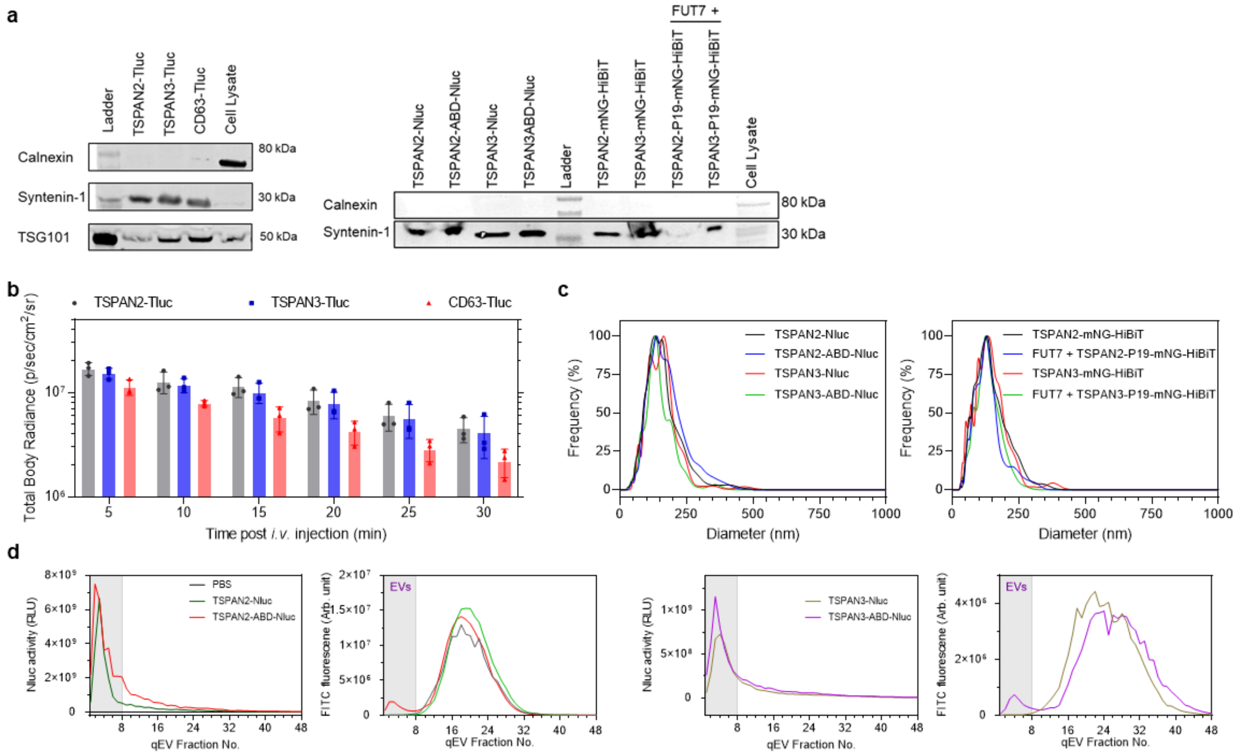

Supplementary Figure 9. Biological activity of engineered EVs. (a) Western blots of engineered EVs showing common EV markers and an exclusion marker. (b) Quantification of total body radiance of mice injected with Tluc labeled-EVs. Mean ± standard deviation of three mice. (c) Size distribution of engineered EVs. (d) Size exclusion chromatography elution profiles of Nluc-labeled EVs after incubating with FITC-HSA conjugates. Nluc activity and FITC fluorescence was measured in each fraction using a plate reader. Source data are provided as a Source Data file.

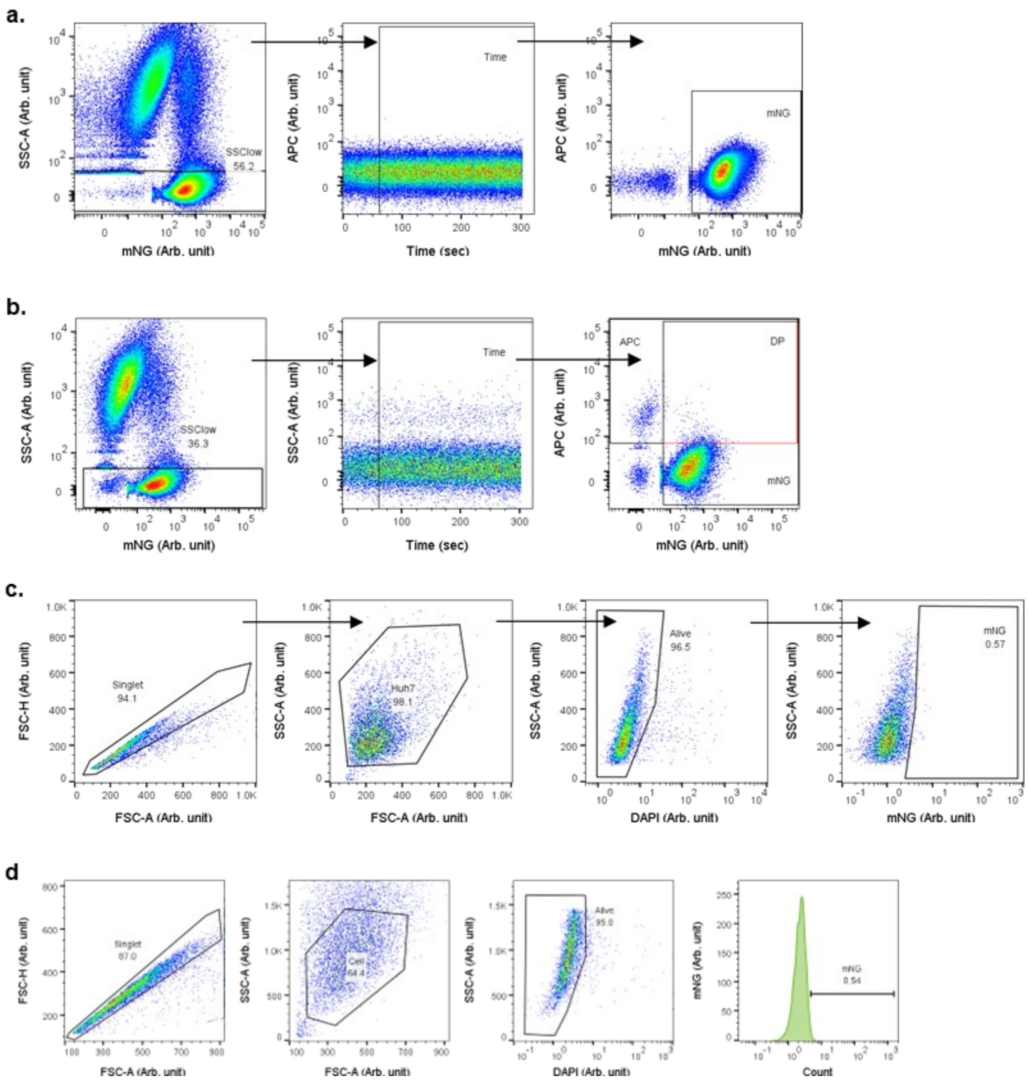

Supplementary Figure 10. Gating strategy in this study. (a) Related to Figure 5d and Supplementary Figure 4. (b) Related to Figure 6e and Supplementary Figure 3. (c) Related to Figure 7b. (d) Related to Figure 7h.
