# Supplementary material for: Identification of scaffold proteins for improved endogenous engineering of extracellular vesicles
Source: Nat Commun. 2023 Aug 7;14:4734. doi: 10.1038/s41467-023-40453-0 (PMC10406850; doi:10.1038/s41467-023-40453-0)
Supplement: Supplementary file 2 — Reporting Summary [file 41467_2023_40453_MOESM2_ESM.pdf]

nature portfolio

Corresponding author(s): Samir El-Andaloussi

Last updated by author(s): Jul 13, 2023

Reporting Summary

Nature Portfolio wishes to improve the reproducibility of the work that we publish. This form provides structure for consistency and transparency in reporting. For further information on Nature Portfolio policies, see our Editorial Policies and the Editorial Policy Checklist.

Statistics

For all statistical analyses, confirm that the following items are present in the figure legend, table legend, main text, or Methods section.

n/a	Confirmed	
☐	☒	The exact sample size (*n*) for each experimental group/condition, given as a discrete number and unit of measurement
☐	☒	A statement on whether measurements were taken from distinct samples or whether the same sample was measured repeatedly
☐	☒	The statistical test(s) used AND whether they are one- or two-sided<br>*Only common tests should be described solely by name; describe more complex techniques in the Methods section.*
☒	☐	A description of all covariates tested
☒	☐	A description of any assumptions or corrections, such as tests of normality and adjustment for multiple comparisons
☐	☒	A full description of the statistical parameters including central tendency (e.g. means) or other basic estimates (e.g. regression coefficient) AND variation (e.g. standard deviation) or associated estimates of uncertainty (e.g. confidence intervals)
☐	☒	For null hypothesis testing, the test statistic (e.g. *F*, *t*, *r*) with confidence intervals, effect sizes, degrees of freedom and *P* value noted<br>*Give P values as exact values whenever suitable.*
☒	☐	For Bayesian analysis, information on the choice of priors and Markov chain Monte Carlo settings
☒	☐	For hierarchical and complex designs, identification of the appropriate level for tests and full reporting of outcomes
☒	☐	Estimates of effect sizes (e.g. Cohen's *d*, Pearson's *r*), indicating how they were calculated

Our web collection on statistics for biologists contains articles on many of the points above.

Software and code

Policy information about availability of computer code

Data collection	Western blotting: LiCor Odyssey Classic Imager.<br>Size distribution and particle concentration: Malvern NanoSight NS500.<br>Electron microscope: Philips CM200-FEG electron microscope.<br>Fluorescence microscope: Nikon A1R confocal microscope.<br>Flow cytometry: Miltenyi MACSQuant Analyzer 10 cytometer; and Luminex Amnis CellStream cytometer.<br>Animal imaging: Perkin-Elmer IVIS Spectrum imager.<br>Plate reader: Promega GloMax 96 Microplate Luminometer; and Molecular Devices SpectraMax i3X
Data analysis	General data analysis: Microsoft Excel (v. 2305), GraphPad Prism (v. 9.2.0).<br>Size distribution and particle concentration: NTA analytical software (v. 3.2)<br>Flow cytometry: FlowJo (v.10.6.2).<br>Proteomics: R (version 4.1.2, 2021-11-01), RStudio (version 2022.07.1+554) and DEP (version 1.16.0), PANTHER 17.0 software.

For manuscripts utilizing custom algorithms or software that are central to the research but not yet described in published literature, software must be made available to editors and reviewers. We strongly encourage code deposition in a community repository (e.g. GitHub). See the Nature Portfolio guidelines for submitting code & software for further information.

Data

Policy information about availability of data

All manuscripts must include a data availability statement. This statement should provide the following information, where applicable:

- Accession codes, unique identifiers, or web links for publicly available datasets
- A description of any restrictions on data availability
- For clinical datasets or third party data, please ensure that the statement adheres to our policy

Source data are provided as a Source Data file. The mass spectrometry proteomics data have been deposited to the ProteomeXchange Consortium via the PRIDE partner repository with the dataset identifier PXD043840.

Human research participants

Policy information about studies involving human research participants and Sex and Gender in Research.

Reporting on sex and gender	Not applicable.
Population characteristics	Not applicable.
Recruitment	Not applicable.
Ethics oversight	Not applicable.

Note that full information on the approval of the study protocol must also be provided in the manuscript.

Field-specific reporting

Please select the one below that is the best fit for your research. If you are not sure, read the appropriate sections before making your selection.

☒ Life sciences ☐ Behavioural & social sciences ☐ Ecological, evolutionary & environmental sciences

For a reference copy of the document with all sections, see nature.com/documents/nr-reporting-summary-flat.pdf

Life sciences study design

All studies must disclose on these points even when the disclosure is negative.

Sample size	No statistical methods were used to predetermine sample size. Sample size was determined based on experimental feasibility, sample availability, necessity to derive definitive conclusions.
Data exclusions	No exclusion criteria applied.
Replication	Cell culture experiments were performed in biological replication. Mouse experiments were done in 3 or 7 per group. All results were from biological replicates.
Randomization	Mice and samples were randomly allocated into study groups.
Blinding	Investigators were not blinded because all results were quantitative and objective. The study did not involve subjective judgement.

Reporting for specific materials, systems and methods

We require information from authors about some types of materials, experimental systems and methods used in many studies. Here, indicate whether each material, system or method listed is relevant to your study. If you are not sure if a list item applies to your research, read the appropriate section before selecting a response.

Materials & experimental systems

n/a	Involved in the study
☐	☒ Antibodies
☐	☒ Eukaryotic cell lines
☒	☐ Palaeontology and archaeology
☐	☒ Animals and other organisms
☒	☐ Clinical data
☒	☐ Dual use research of concern

Methods

n/a	Involved in the study
☒	☐ ChIP-seq
☐	☒ Flow cytometry
☒	☐ MRI-based neuroimaging

Antibodies

Antibodies used

anti-TSG101 [Abcam, ab30871];
anti-Calnexin [ThermoFisher, PA5-19169];
anti-Syntenin-1 [Origene, TA504796];
anti-CD81 [SantaCruz, sc-9158];
anti-β-actin [Sigma, A5441];
anti-TSPAN2 [FAB7876R; R&D systems];
anti-CD9 [Miltenyi, 130-103-956];
anti-CD63 [Miltenyi, 130-100-182];
anti-CD81 [Beckman Coulter, A87789]
Goat-anti-mouse/rabbit secondary antibody: Licor: 925-68070, 926-68071, 926-32210, 926-32211

Validation

All antibodies are commercially available and tested by manufacturers with detailed specificity described on their websites. Our lab have used those antibodies on a routine basis.

anti-TSG101 [Abcam, ab30871]; Suitable for: WB, ICC/IF, IHC-P; Reacts with: Mouse, Rat, Human
https://www.abcam.com/products/primary-antibodies/tsg101-antibody-ab30871.html

anti-Calnexin [ThermoFisher, PA5-19169]; Suitable for: WB, ICC/IF, IHC; Reacts with: Mouse, Human
https://www.thermofisher.com/antibody/product/Calnexin-Antibody-Polyclonal/PA5-19169

anti-Syntenin-1 [Origene, TA504796]; Suitable for: WB, IHC; Reacts with: Mouse, Rat, Human
https://www.origene.com/catalog/antibodies/primary-antibodies/ta504796/syntenin-sdcbp-mouse-monoclonal-antibody-clone-id-oti2h6

anti-CD81 [SantaCruz, sc-9158]; Suitable for: WB, IHC; Reacts with: Mouse, Rat, Human
https://www.scbt.com/p/cd81-antibody-h-121

anti-β-actin [Sigma, A5441]; Suitable for: WB, IHC, IF; Reacts with: Mouse, Rat, Human, et al.
https://www.sigmaaldrich.com/SE/en/product/sigma/a5441

anti-TSPAN2 [FAB7876R; R&D systems]; Suitable for: FC; Reacts with: Human
https://www.rndsystems.com/products/human-tspan2-alexa-fluor-647-conjugated-antibody-822509_fab7876r

anti-CD9 [Miltenyi, 130-103-956]; Suitable for: FC; Reacts with: Human
https://www.miltenyibiotec.com/SE-en/products/cd9-antibody-anti-human-sn4-c3-3a2.html

anti-CD63 [Miltenyi, 130-100-182]; Suitable for: FC; Reacts with: Human
https://www.miltenyibiotec.com/US-en/products/cd63-antibody-anti-human-h5c6.html

anti-CD81 [Beckman Coulter, A87789]; Suitable for: FC; Reacts with: Human
https://www.mybeckman.se/en/reagents/coulter-flow-cytometry/antibodies-and-kits/single-color-antibodies/cd81/a87789

Eukaryotic cell lines

Policy information about cell lines and Sex and Gender in Research

Cell line source(s)

HEK-293T (ATCC, CRL-3216),
Huh-7 (XenoTech, JCRB0403)
HUVEC (ATCC, CRL-4053)
TCMK-1 (ATCC, CCL-139)
MSCs (ATCC, PCS-500-010)
Freestyle 293-F (ThermoFisher, R79007)

Authentication

Cell lines were authenticated by morphology in our lab.

Mycoplasma contamination

Mycoplasma tests were performed every month according out our lab's routine and returned negative.

Commonly misidentified lines (See ICLAC register)	No commonly misidentified cell lines were used.

Animals and other research organisms

Policy information about studies involving animals; ARRIVE guidelines recommended for reporting animal research, and Sex and Gender in Research

Laboratory animals	Female NMRI mice were bought from Charles River (around 20 g of body weight and 3 weeks of age) and housed in our animal facility for at least one week before use according to standard routines (temperature: 20-22°C, humidity: 45-55%, dark/light cycle: 12/12 h).
Wild animals	The study did not involve wild animals.
Reporting on sex	Only female mice were used in this study because of lower housing cost.
Field-collected samples	The study did not involve samples collected from the field.
Ethics oversight	All animal experiments were performed in accordance with ethical permission granted by Swedish Jordbruksverket (permit No.13849-2020).

Note that full information on the approval of the study protocol must also be provided in the manuscript.

Flow Cytometry

Plots

Confirm that:

- [x] The axis labels state the marker and fluorochrome used (e.g. CD4-FITC).
- [x] The axis scales are clearly visible. Include numbers along axes only for bottom left plot of group (a 'group' is an analysis of identical markers).
- [x] All plots are contour plots with outliers or pseudocolor plots.
- [x] A numerical value for number of cells or percentage (with statistics) is provided.

Methodology

Sample preparation	Flow cytometry for cells<br>Huh-7 cells were seeded at 3e4 per well in 96-well plate and incubated overnight. The cells were treated with HiBiT-mNG-labeled EVs for 8 hr. and then trypsinized and resuspended in 100 µL of PBS containing 2% FBS. 4′,6-diamidino-2-phenylindole (DAPI) staining was added to all samples to exclude dead cells from the analysis.<br>Flow cytometry for multiplex beads<br>MACSPlex Exosome Kit (Miltenyi Biotec; 130-108-813) was used to characterize the surface protein composition of EVs following manufacturers' instructions. In brief, EVs (1×109 in 120µL) were incubated with 15µL of MACSPlex exosome capture beads overnight in wells of a pre-wet and drained MACSPlex 96-well 0.22µm filter plate at room temperature. The beads were rinsed with 200µL MACSPlex buffer and detected after staining with APC-conjugated antibody mixture (anti-CD9/CD63/CD81) or AF647-conjugated anti-TSPAN2 antibodies (FAB7876R; R&D systems) for 1h at room temperature. Next, the samples were rinsed twice, resuspended and analyzed using MACSQuant Analyzer 10 flow cytometer. FlowJo (v.10.6.2) was used to analyze data. Median fluorescence intensities (MFIs) for all 39 capture bead subsets were background-corrected by subtracting the respective values from matched non-EV-containing buffer controls and normalized to the beads with the highest level.<br><br>Flow cytometry for extracellular vesicles<br>The HiBiT-mNG-tagged EVs were analyzed using the side scatter laser (SSC; 40% of maximum power) and 488 nm lasers (maximum power) at the Amnis CellStream instrument (Luminex, US). All data was acquired with low flow rate of 3.44 µL/min. SSC and mNG signals were collected in channel 1 (773 ± 56 nm filter) and channel C3 (528 ± 46 nm filter), respectively. Dulbecco's PBS pH 7.4 (Gibco, 14190-136) was used without further filtration as sheath fluid.
Instrument	Cells: MACSQuant Analyzer 10 cytometer (Miltenyi, Germany). EVs: Luminex Amnis CellStream cytometer.
Software	FlowJo software (version 10.6.2)
Cell population abundance	For cells: the percentage of parent population and mean fluorescence intensity of single alive cells<br>For beads: mean fluorescence intensity<br>For EVs: concentration oand mean fluorescence intensity f fluorescent particles
Gating strategy	For cells, single (FSC-A vs FSC-H) > cells > alive (DAPI-negative) > mNG;<br>For beads: single (FSC-A vs FSC-H) > beads > APC (refer to our previous publication: doi.org/10.1002/jev2.12238);<br>For EVs: SSC-low > Time > mNG/APC (refer to our previous publication: doi.org/10.1002/jev2.12238).

- [x] Tick this box to confirm that a figure exemplifying the gating strategy is provided in the Supplementary Information.
